# Proteomic study of left ventricle and cortex in rats after myocardial infarction

**DOI:** 10.1038/s41598-024-56816-6

**Published:** 2024-03-22

**Authors:** Mengli Chang, Huanhuan Wang, Yuxin Lei, Hongjun Yang, Jing Xu, Shihuan Tang

**Affiliations:** 1 Institute of Chinese Materia Medica, China Academy of Chinese Medical Sciences, Beijing, 100700 China; 2 Experimental Research Center, China Academy of Chinese Medical Sciences, Beijing, 100700 China

**Keywords:** Myocardial infarction, Neuroinflammation, IBA-1, DIA, Heart–brain interaction, Immunology, Diseases, Cardiovascular diseases

## Abstract

Myocardial infarction (MI) induces neuroinflammation indirectly, chronic neuroinflammation may cause neurodegenerative diseases. Changes in the proteomics of heart and brain tissue after MI may shed new light on the mechanisms involved in neuroinflammation. This study explored brain and heart protein changes after MI with a data-independent acquisition (DIA) mode proteomics approach. Permanent ligation of the left anterior descending coronary artery (LAD) was performed in the heart of rats, and the immunofluorescence of microglia in the brain cortex was performed at 1d, 3d, 5d, and 7d after MI to detect the neuroinflammation. Then proteomics was accomplished to obtain the vital proteins in the heart and brain post-MI. The results show that the number of microglia was significantly increased in the Model-1d group, the Model-3d group, the Model-5d group, and the Model-7d group compared to the Sham group. Various proteins were obtained through DIA proteomics. Linking to key targets of brain disease, 14 proteins were obtained in the brain cortex. Among them, elongation of very long chain fatty acids protein 5 (ELOVL5) and ATP-binding cassette subfamily G member 4 (ABCG4) were verified through western blotting (WB). The results of WB were consistent with the proteomics results. Therefore, these proteins may be related to the pathogenesis of neuroinflammation after MI.

## Introduction

Cardiovascular and cerebrovascular diseases affect each other. The pathological mechanism between the heart and the brain is complicated. Patients with Myocardial infarction (MI) still have a risk of getting cerebral infarction and depression after admission to the hospital, which probably contributes to an augmented mortality after MI^1–4^. There has been an increased focus on the pathogenesis in the heart and brain after MI. Various factors contribute to their pathogenesis, such as regulating of the sympathetic nervous system, releasing catecholamines, brain-gut axis, and hypothalamic pituitary adrenal axis^5–7^. An inflammatory reaction is one of the most concerning mechanisms in the cross-talk between the heart and the brain.

Inflammatory response is two-sided and contributes to the changes in the left ventricular (LV) and brain after MI. On the one hand, immunological pathways are dysregulated after MI and there is an excessive inflammatory response which in turn activates myocardial fibrosis (MF) in the infarcted tissue to promote myocardial remodeling, thereby contributing to cardiac dysfunction^8–11^. On the other hand, inflammation contributes to the pathogenesis of neurological disorders induced mostly by the activation of glial cells^12–14^. Microglia are activated from the resting state to M1/M2 polarization, signifies the pro-inflammatory or anti-inflammatory state in the brain^12^, which contributes to the development of neurogenerative diseases such as Alzheimer's disease (AD) and Parkinson's disease (PD)^15–17^. Neuroinflammation is characterized by the activated microglia in the brain after MI^7,18^. The elevation expression of translocator protein (18kDa) (TSPO) predicts cardiac inflammatory response in the infarcted area of the LV paralleled by neuroinflammation in the brain after MI, which in turn contributes to cardiac and brain dysfunction^19,20^. However, how neuroinflammation develops in the brain after MI remains unclear. Proteomics technology is widely used for studying cardiovascular and neurological diseases. Using targeted plasma proteomics to study specific pathways: in contrast to a clear interleukin-6 signal in high CRP patients, neutrophil-signaling-related proteins were associated with recurrent atherosclerotic cardiovascular disease events in low CRP patients. These specific pathways based on the targeted plasma proteomics helped to establish a proteome-based risk model to predict recurrent atherosclerotic cardiovascular disease events in the secondary prevention of cardiovascular disease^21^. PYGL, ASPH, and CD45 were identified as spatial markers for tumor boundary, and protein networks of immune response-driven, spatially-organized were revealed based on the quantitative proteomics of tissue^22^. Longitudinal plasma proteomics helped to reveal the biomarkers of alveolar-capillary barrier disruption in COVID-19 patients and assess the effects of imatinib treatment^23^.

**Figure 1:**
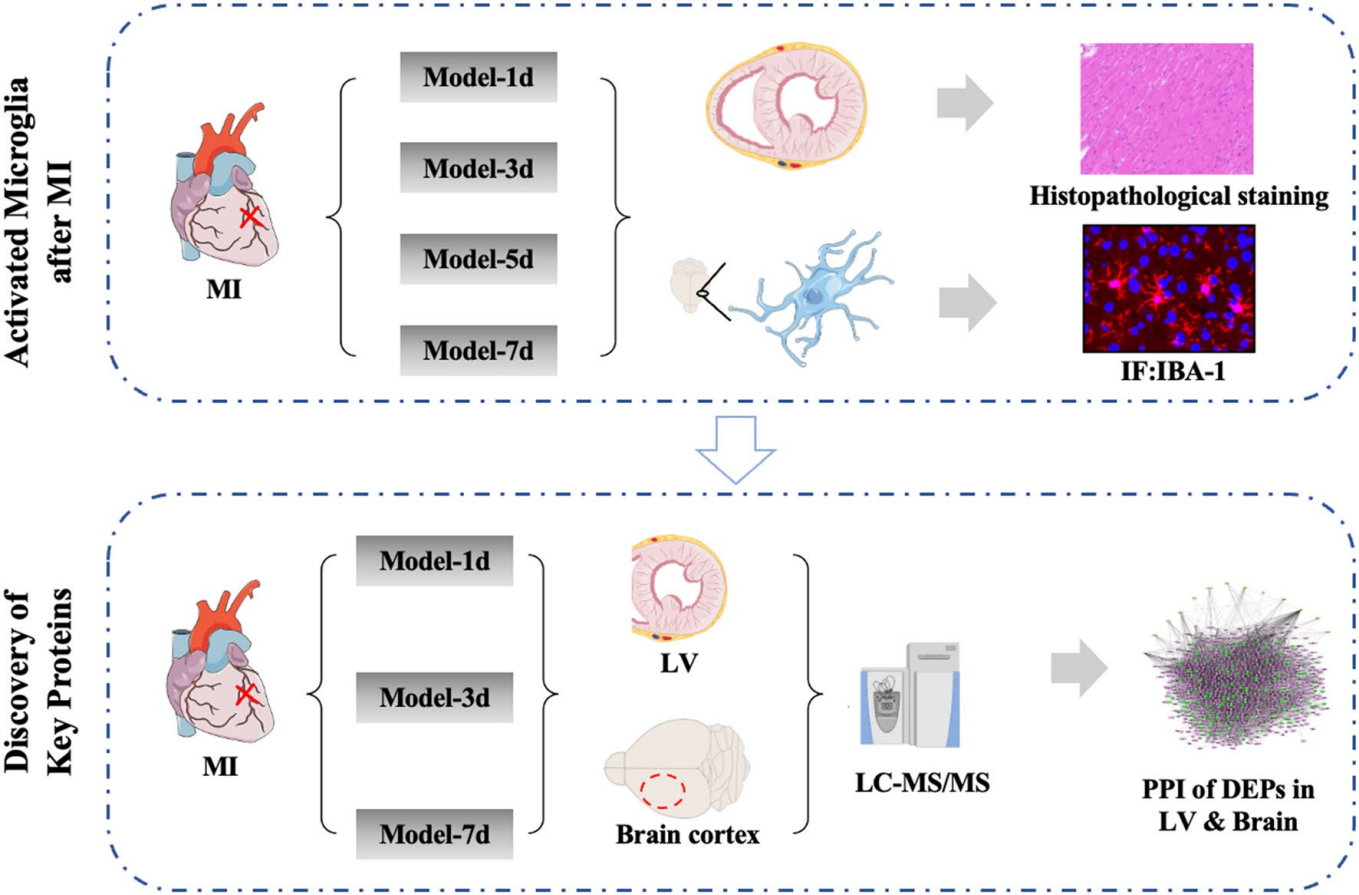
Overall design of the study. MI: Myocardial infarction, LV: Left ventricular, DEPs: Differentially expressed proteins, IF: Immunofluorescence, Model-1d: 1 day after myocardial infarction, Model-3d: 3 days after myocardial infarction, Model-5d: 5 days after myocardial infarction, Model-7d: 7 days after myocardial infarction. IBA-1: ionized calcium-binding adaptor molecule-1, LC-MS/MS: liquid chromatography-mass spectrometry/mass spectrometry, PPI: Protein–protein interaction.

In this study, immunofluorescence of microglia in the brain sections was performed to assess brain inflammation. Data-independent acquisition (DIA) mode proteomics of the LV and the brain cortex was applied to discover the possible key proteins that might mediate myocardial injury and neuroinflammation in the brain (Fig. 1). The study aimed to provide an objective results of proteomics in the LV and the brain cortex after MI as well as share a strategy to study the disease complications.

## Results

### Histopathological changes of heart and brain after MI

**Figure 2:**
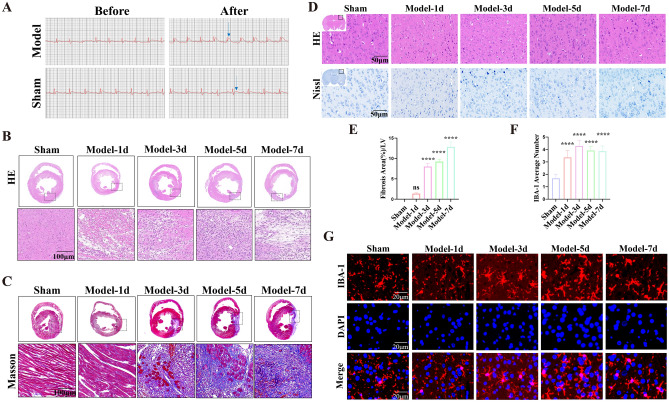
Histopathological staining at 1d, 3d, 5d, and 7d after MI. (**A**) Representative photographs of ECG information of Sham and Model rats. The blue arrow points to the ST segment. (**B**) Representative photographs of H&E staining of heart sections after MI. (**C** and **E**) Representative photographs and quantitative data of Masson’s trichrome staining of heart sections. (**D**) H&E staining and Nissl staining of brain sections at different time points after MI. (**F**) Quantitative data of the expression of IBA-1 in the brain cortex at different time points after MI. (****p < 0.0001 vs. Sham group). (**G**) Representative photographs of immunofluorescence labeling of microglia in the brain cortex at different time points after MI. Merge represents IBA-1 overlaps with DAPI, which was considered to be microglia. The higher the number of microglia, the more severe the neuroinflammation in the cortex. Model-1d means one day after MI, Model-3d means three days after MI, Model-5d means five days after MI, and Model-7d means seven days after MI.

A total of 21 rats died during the surgery and 8 rats failed to ligate the LAD. The elevated ST segment on electrocardiogram (ECG) was observed after ligation of LAD (Fig. 2A), indicating the MI model was established successfully. H&E staining was performed to detect the pathological changes in LV after MI (Fig. 2B). Infiltration of inflammatory cells and disorganization of muscle fibers were observed in the Model group when compared with the Sham group post-ligation for 1d, 3d, 5d, and 7d. Moreover, hemorrhage, cardiac myocyte swelling and fragmentation, myocardial nuclei loss, and vascular proliferation occurred at 3d and 5d after LAD ligation in rats in the Model group compared with the Sham group. MF occurred gradually after ligation (Fig. 2C) based on Masson staining. Collagen fiber area increased at 3d after MI in the Model group, and also significantly increased at 5d, as well as 7d after MI when compared to the Sham group (Fig. 2E). H&E and Nissl staining were performed to observe histopathological changes in the brain tissue after MI. There was no necrosis, degeneration, or autophagy phenomenon in the brain. The structure of the brain tissue was normal, and the abundant neuronal cells were arranged neatly both in the Sham group and the Model group. Nissl staining displayed plenty of nissl bodies with intact structure and abundant cytoplasm without degeneration or deepening of staining (Fig. 2D). These results suggested that the brain shows no obvious pathological changes after MI.

### The number of IBA-1-positive microglia was increased after MI

Neuroinflammation is a fundamental pathological mechanism in the brain diseases, such as neurodegenerative disorders and TBI. Microglia are essential to the development of neurons and the operation of immune surveillance functions^19,24,25^. Studies have shown that activated microglia induced by MI lead to neuroinflammation in the brain early^26^. The ionized calcium-binding adaptor molecule-1 (IBA-1) is specifically expressed in the microglia in the brain and it has been widely used as a microglial marker^27^. In this study, the number of IBA-1-positive microglia was significantly increased in the brain cortex (Fig. 2F). They had larger cell bodies and became amoeboid morphology (Fig. 2G) at 1d, 3d, 5d, and 7d after MI in the Model group when compared with the Sham group. These results indicated that the microglial activation occurred in the brain cortex after MI early.

### Proteome changes in the heart and brain after MI

**Figure 3:**
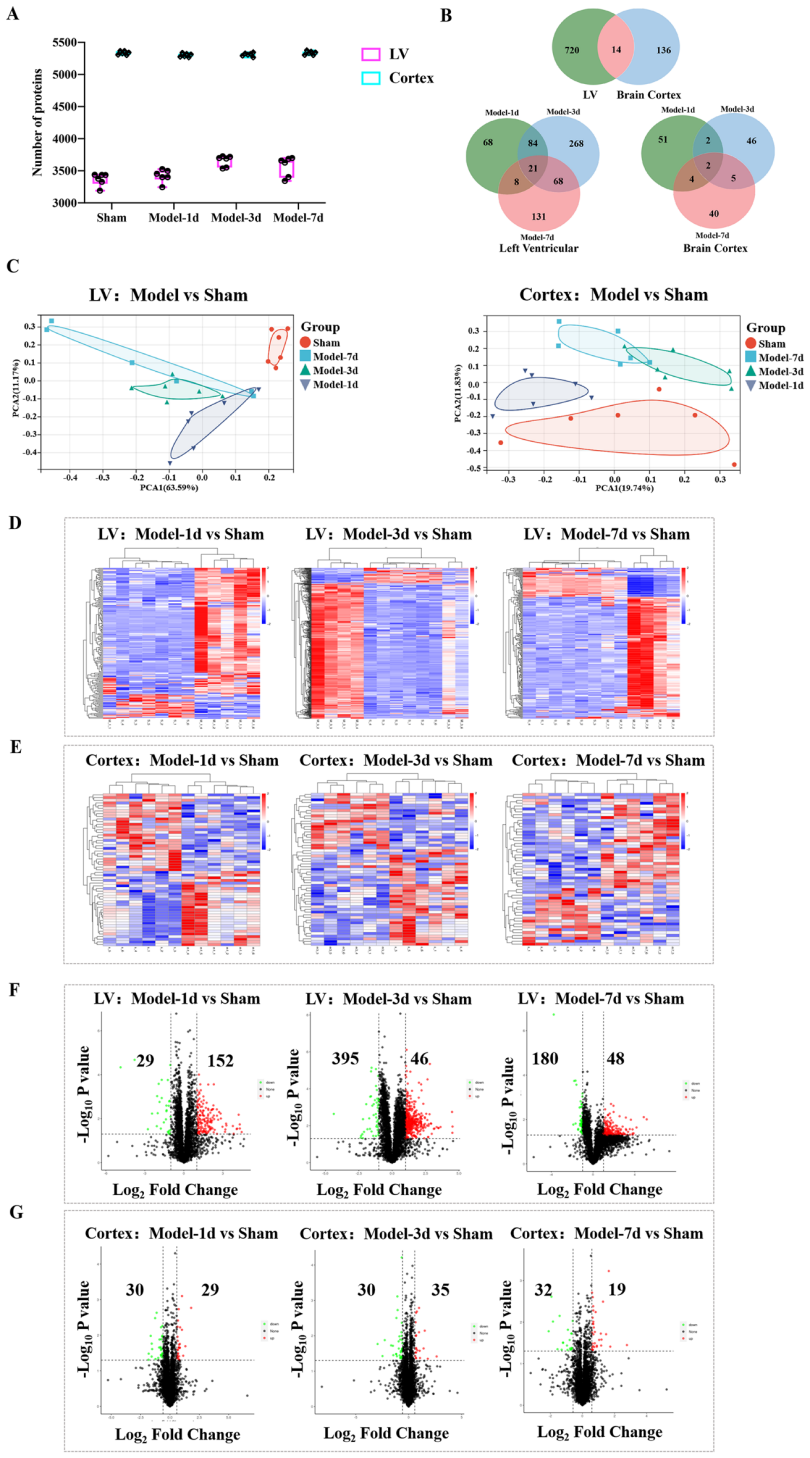
Proteomic Identification Results in the LV and the brain cortex. (**A**) Protein numbers identified in the LV and the brain cortex of each rat. (**B**) Number of DEPs detected in the LV and brain cortex. The top Venn diagram shows the intersection DEPs of the LV and the brain cortex, while the other two Venn diagrams demonstrate intersection DEPs at 1d, 3d, and 7d post-MI in the LV and the brain cortex. (**C**) PCA and consensus clustering in LV and brain cortex, which reflects overall protein differences between each group of samples and the magnitude of variability between samples within a group. (**D**) Heat maps show DEPs in the LV at different time points after MI. (**E**) Heat maps show DEPs in the brain cortex at different time points after MI. (**F**) Volcano plots show the number of up and down proteins regulated in the LV at different time points after MI. (**G**) Volcano plots show the number of up and down proteins regulated in the brain cortex at different time points after MI. PCA plots, Volcano plots, and Venn plots were all analyzed in the Sangerbox analysis platform online (http://vip.sangerbox.com/home.html).

According to the Statistical data results of IBA-1 staining in the brain cortex, the LV tissue and the brain cortex of Model-1d, Model-3d, and Model-7d were subject to a DIA mode proteomics analysis to detect the protein changes after MI. The distribution plot of unique peptide numbers in identified proteins demonstrated that the curve increased slowly as the number of Unique peptides increased, thereby indicating that large amounts of reliable proteins are identified (Fig. S1). The normalization method of proteomic data was Median normalization. The results revealed that proteins were expressed stably in the LV as well as the brain cortex (Fig. 3A). A total of 4 154 proteins were identified in the LV tissue and 5 648 proteins were identified in the brain cortex. FC ≥ 1.5 and *p*-value < 0.05 were considered upregulated, and FC ≤ 0.50, and *p-*value < 0.05 were considered downregulated. Principal Component Analysis (PCA) analysis (Fig. 3C, Fig. S2A) of DEPs results showed the obvious distinction between the Model-1d group, Model-3d group, Model-7d group, and Sham group, indicating that the different expressing modes of proteins between the Sham and the Model group. Moreover, DEPs in the Model groups at different time points also had an obvious separation, which indicated that the proteins have changed both in the LV and the brain cortex. Based on the unsupervised mode, the DEPs in the LV and the brain cortex were analyzed and presented by heatmaps. DEPs in every Model group and Sham group in the LV (Fig. 3D) and the brain cortex (Fig. 3E) could be grouped into two subclusters.

734 DEPs were identified in the LV and 150 DEPs were identified in the brain cortex. (Fig. 3F) Including 181 DEPs in the LV 1d after MI (152 DEPs upregulated and 29 DEPs downregulated), 441 DEPs in the LV 3d after MI (395 DEPs upregulated and 46 DEPs downregulated), and 228 DEPs in the LV 7d after MI (180 DEPs upregulated and 48 DEPs downregulated). While, in the brain cortex (Fig. 3G), 59 proteins have changed 1d after MI, including 30 DEPs upregulated and 29 DEPs downregulated. 55 proteins have changed 3d after MI, including 20 DEPs upregulated and 35 DEPs downregulated. 51 DEPs were identified in the brain cortex 7d after MI, including 32 DEPs upregulated and 19 DEPs downregulated. 105 common DEPs were identified at both 1d and 3d after MI, 89 DEPs at both 3d and 7d after MI, and 21 DEPs at 1d, 3d, and 7d in the LV after MI. 4 common DEPs were identified at both 1d and 3d after MI, 7 DEPs at both 3d, and 7d after MI, and 2 DEPs at 1d, 3d, and 7d in the LV after MI (Fig. 3B). These results suggested that plentiful proteins were changed in the LV and the brain cortex after MI.

### GO and KEGG enrichment of DEPs in the LV and the brain cortex

GO and KEGG analyses are shown in Fig. S2B, S2C. The DEPs in the LV 1d after MI are involved in the glycosaminoglycan metabolic process, hyaluronan metabolic process, and lipoprotein metabolic process, as well as platelet activation. The DEPs in the LV 3d after MI are involved in blood coagulation, platelet activation, and cell redox homeostasis, which is located in the endoplasmic reticulum and related the oxidative stress (OS) and inflammatory response. The top of GO-MF is calcium ion binding. The top GO-BP of DEPs in the LV 7d after MI are immune response as well as antigen processing and presentation. They are seen predominantly in the nucleus. The DEPs in the brain cortex 1d post-MI are mostly related to cellular iron ion homeostasis and proteolysis. The top GO-BPs of DEPs in the brain cortex 3d after MI are the dopamine receptor signaling pathway, copper ion transport, and neuropeptide signaling pathway, etc. The top BPs of DEPs in the brain cortex 7 days after MI are chloride transport and mannose metabolic process. Complement and coagulation cascades are the top 1 pathway that enriched in LV both at 1d and 3d post-MI. The DEPs in LV 7d post-MI are related to pathways like phagosome, serotonergic synapse, and cell adhesion molecules. Pathways enriched at 1d post-MI in the brain cortex are necroptosis, ferroptosis, and Neuroactive ligand-receptor interaction. Focal adhesion and platelet activation are the top two pathways that are enriched 3d post-MI in the brain cortex. Ether lipid metabolism, glycerophospholipid metabolism, and purine metabolism which are involved in energy metabolism are mostly enriched in the brain cortex 7d post-MI.

### DEPs that are associated with different pathologic processes in the LV after MI

#### Myocardial injury

**Figure 4:**
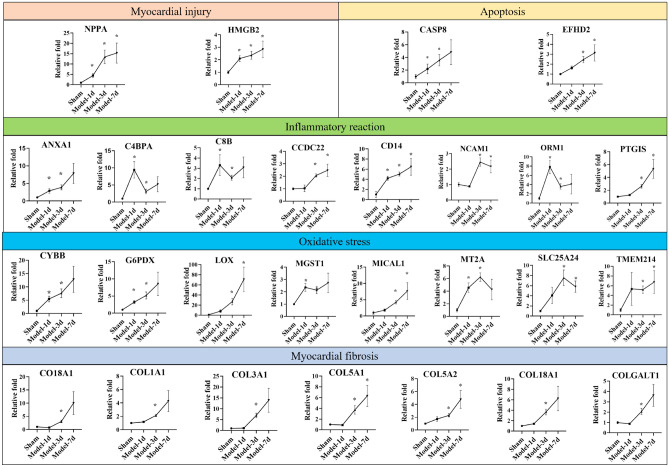
The expression of DEPs related to myocardial injury after MI. The abscissa represents ligation time, the ordinate represents protein expression (All of the expression values divided by the average expression value of the Sham group). * *p* < 0.05, Model vs Sham group.

MI leads to myocardial injury, fibrosis, generation of high levels of reactive oxygen species (ROS), and inflammatory response. Proteins that are associated with MI, such as the atrial natriuretic peptide (NPPA, FC = 2.9274, FC = 8.9812, FC = 10.2193, *p* < 0.05, respectively at 1, 3, and 7d), monocyte differentiation antigen (CD14, FC = 2.8035, FC = 3.3507, FC = 4.30926965, *p* < 0.05, respectively at 1, 3, and 7d) and high mobility group protein B2(HMGB2, FC = 2.1010, FC = 2.3597, FC = 2.8397,* p* < 0.05, respectively at 1, 3 and 7d) were upregulated after MI in the heart tissue when compared with the Sham group (Fig. 4). These results indicated that myocardial injury occurred and deteriorated as the extend of ligation time.

#### Inflammatory response

DEPs that are related to inflammatory response were changed dynamically, such as complement and coagulation cascades: complement component C8 beta chain (C8B, FC = 3.9686, FC = 2.0851, *p* < 0.05, respectively at 1d and 3d) and C4b-binding protein alpha chain (C4BPA, FC = 9.4362, FC = 3.1017, *p* < 0.05, respectively at 1d and 3d). Arachidonic acid metabolism: prostacyclin synthase (PTGIS, FC = 2.6292, FC = 5.31591, *p* < 0.05, respectively at 3d and 7d). Member of the immunoglobulin superfamily: Neural cell adhesion molecule 1(NCAM1, FC = 2.4554, FC = 2.1674, *p* < 0.05, respectively at 3d and 7d). Transport protein in the bloodstream: alpha-1-acid glycoprotein (ORM1, FC = 6.5262, FC = 2.9996, *p* < 0.05, respectively at 1d and 3d). Lysosome transport: coiled-coil domain-containing protein 22 (CCDC22, FC = 2.0678, FC = 2.4823, *p* < 0.05, respectively at 3d and 7d), signaling and cellular processes: Annexin A1(ANXA1, FC = 2.8877, FC = 3.8783, *p* < 0.05, respectively at 1d and 3d) also have a significant expression change.

#### Oxidative stress

OS is one of the most crucial pathological links. Proteins related to the occurrence of OS and OS protection were both identified. Genetic variants of cytochrome b-245 beta chain (CYBB, FC = 3.6433, FC = 5.0831, *p* < 0.05, respectively at 1d and 3d) and Metallothionein (MT2A, FC = 4.5625, FC = 6.2489, *p* < 0.05, respectively at 1d and 3d) promote OS and lead to cell injury^28,29^. Increased OS in the endoplasmic reticulum induces overexpression of transmembrane protein 214 (TMEM214, FC = 3.4609, FC = 5.3989, *p* < 0.05, respectively at 3d and 7d) and induces apoptosis^30^. Glucose-6-phosphate 1-dehydrogenase (G6PDX, FC = 3.2222, FC = 5.1741, *p* < 0.05, respectively at 1d and 3d), Glutathione transferase (MGST1, FC = 2.3553, FC = 2.1340, *p* < 0.05, respectively at 1d and 3d) and [F-actin]-monooxygenase MICAL1 (MICAL1, FC = 2.1243, FC = 4.5762, *p* < 0.05, respectively at 3d and 7d) have anti-oxidant activity and are critical for protecting the cells from OS^31–33^, Solute Carrier Family 25 Member 24 (SLC25A24, FC = 7.5500, FC = 5.8037, *p* < 0.05, respectively at 3d and 7d) reduces cell death by decreasing OS caused by Ca(2 +) overload^34^.

#### Myocardial fibrosis

Protein expression of the fibrillar collagens are increased after MI, such as Collagen alpha-1(V) chain (COL5A1, FC = 3.7221, FC = 6.3115,* p* < 0.05, respectively at 3d and 7d), Collagen alpha-1(V) chain (COL5A2, FC = 2.2914, FC = 4.7711,* p* < 0.05, respectively at 3d and 7d), Collagen type XVIII alpha 1 chain (COL18A1, FC = 3.6638,* p* < 0.05, respectively at 3d), Collagen type VIII alpha 1 chain (COL8A1, FC = 2.5453,* p* < 0.05, respectively at 3d). This suggested that myocardial injury worsens due to ischemia. Dynamic changes of Protein-lysine 6-oxidase (LOX, FC = 4.0842, FC = 35.3411, *p* < 0.05, respectively at 1d and 7d) protein expression are considered to be related with tissue fibrosis, too^35^.

#### Myocardial necrosis

Caspase-8(CASP8, FC = 2.1905, FC = 2.8460, p < 0.05, respectively at 1d and 3d) and EF-hand domain-containing protein D2(EFHD2, FC = 2.4515, FC = 3.1388, *p* < 0.05, respectively at 3d and 7d) are significantly upregulated in the heart. This in turn may lead to myocardial necrosis. This indicates that cardiomyocyte damage is aggravated and towards death gradually during ischemia time.

**Figure 5:**
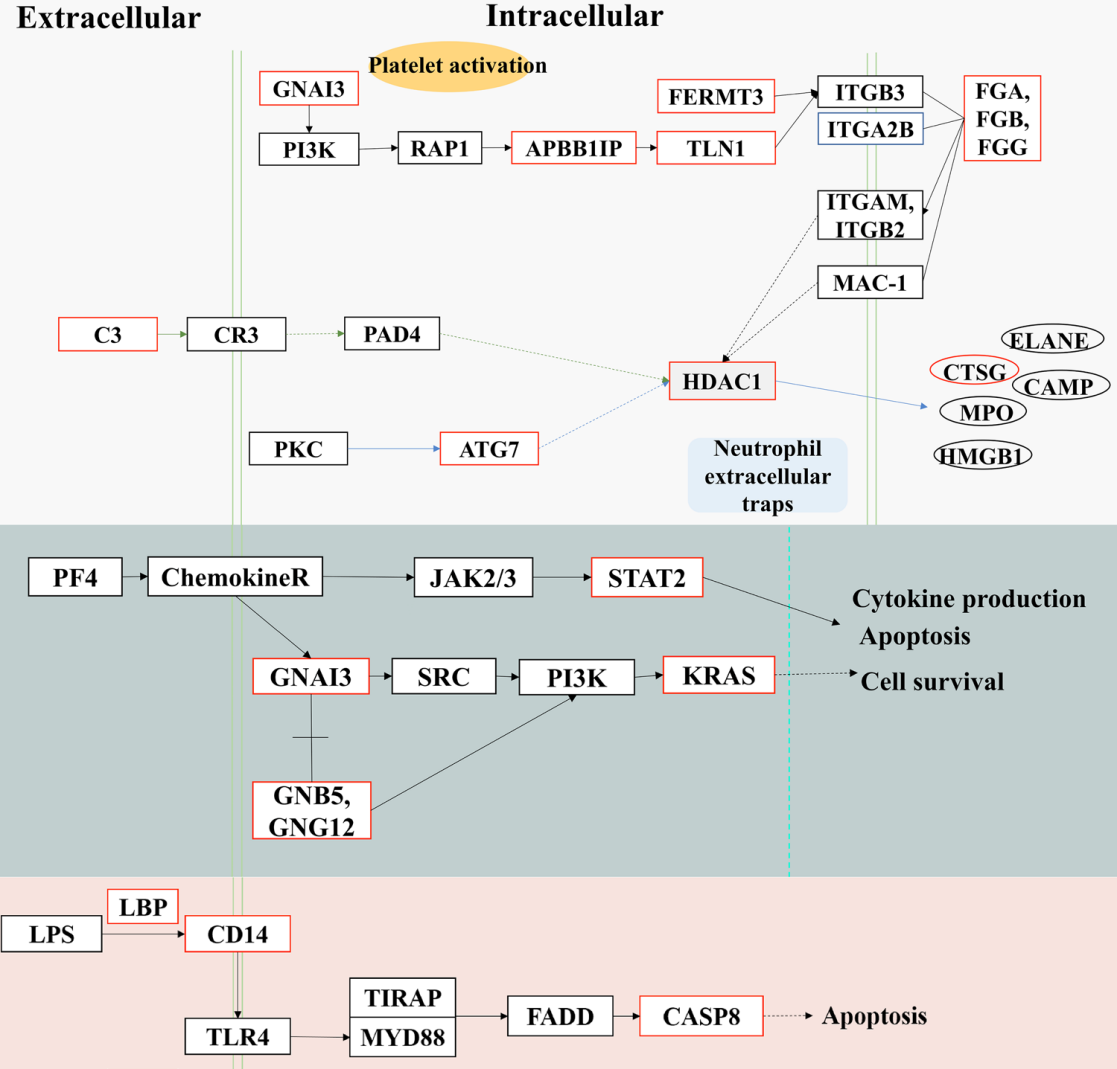
Altered pathways of DEPs in the LV after MI. Proteins with red rectangles represent upregulation based on DIA analysis. Dotted lines between rectangular boxes represent indirect action, solid lines represent direct action.

DEPs in the LV (MI-1d, MI-3d, MI-7d) were performed to KEGG enrichment analysis to recognize the pathways after MI (Fig. 5). These results demonstrated that the Fibrinogen alpha chain (FGA), Fibrinogen beta chain (FGB), and Fibrinogen gamma chain (FGG) are upregulated in activated platelets via an ITGB3-dependent pathway. They also promote the formation of neutrophil extracellular traps (NETs) and the release of cathepsin G(CTSG). Cytokine production is activated via V-Ki-ras2 Kirsten rat sarcoma viral oncogene homolog (KRAS) and Signal transducer and activator of transcription (STAT2). There is the activation of the LPS-CD14-TLR4 pathway, which leads to apoptosis via the activation of CASP8.

### DEPs that are associated with brain diseases

**Figure 6:**
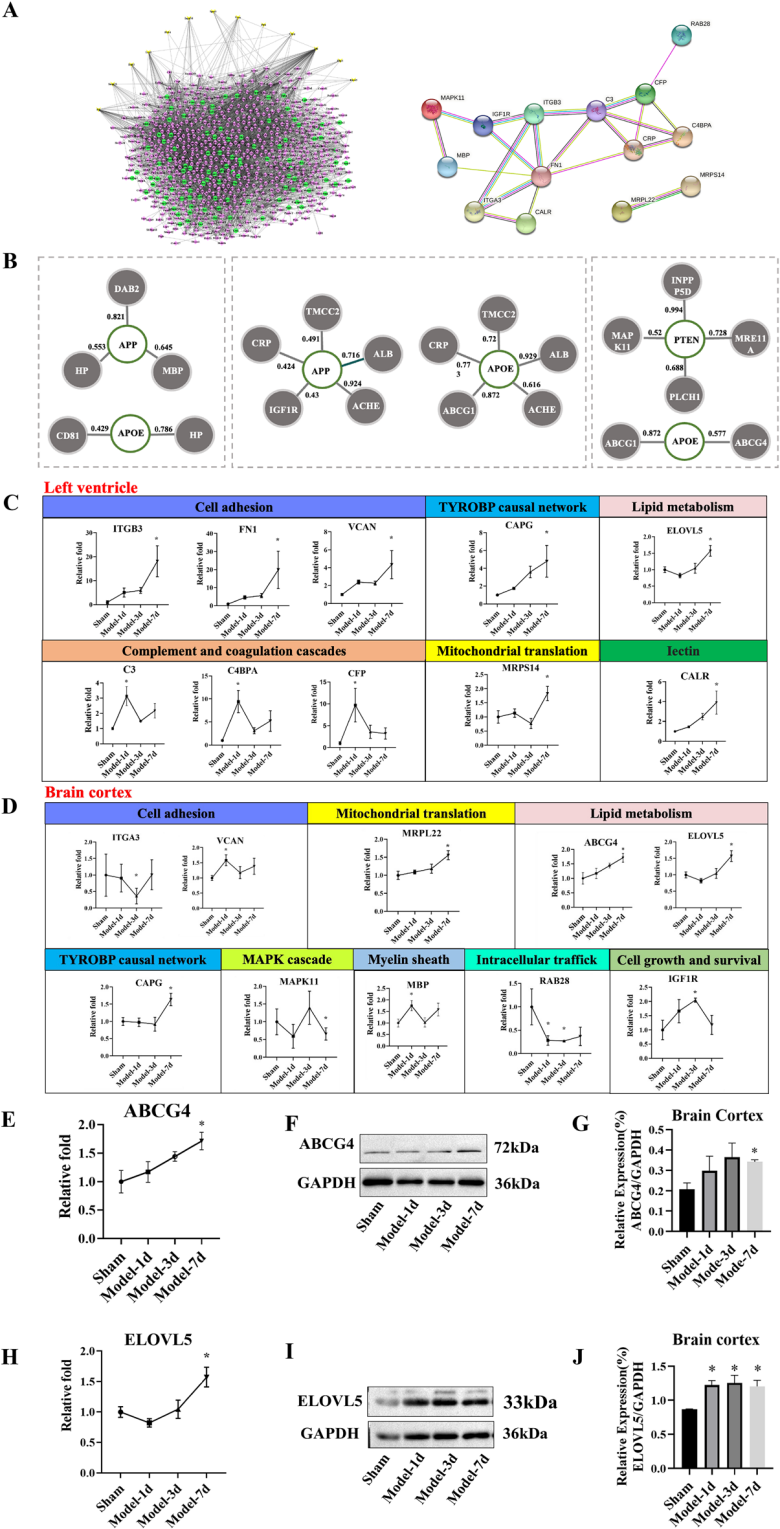
Proteins associated with brain diseases. (**A**) The PPI of DEPs in the LV and the brain cortex. (**B**) DEPs related to APP, PTEN, and APOE at 1 d, 3 d, and 7 d after MI according to the combined score based on the STRING online database. (**C**, **D**) DEPs with significant changes in protein expression at all time points in the LV and brain cortex after MI. (**E**, **H**) Protein expression of ABCG4 and ELOVL5 according to proteomics results. (**F**, **I**) Representative photographs of western blotting results of ELOVL5 and ABCG4. (**G**, **J**) Quantitative data of western blotting results of ELOVL5 and ABCG4. * *p* < 0.05, Model vs Sham group.

The PPI of DEPs in the LV and the brain was formed (Fig. 6A, the left one). 14 DEPs were both in the LV and the brain cortex, including Lymphocyte cytosolic protein 1 (LCP1)), C-reactive protein (CRP), Phosphatidylinositol-3,4,5-trisphosphate 5-phosphatase (INPP5D), Macrophage-capping protein (CAPG), Versican core protein (VCAN), Haptoglobin (HP), Disabled homolog 2 (DAB2), Ferritin light chain 1 (FTL1), Synaptosomal-associated protein 23 (SNAP23), Phosphoinositide phospholipase C (PLCH1), SPCS3 (Signal peptidase complex subunit 3), Retinol-binding protein 1 (RBP1), Albumin (ALB) and Elongation of very long chain fatty acids protein 5 (ELOVL5).

To search for the link between DEPs and brain diseases, the retained DEPs were built a relationship with core targets of cognitive dysfunction (Table S1), adhesion spot (Table S2), energy metabolism (Table S3), and neuroinflammation (Table S4) from GeneCards database (https://www.genecards.org/). We considered only the top 25 targets to ensure that the collected data was reliable. All the targets were collected and compared with DEPs in this study. The result demonstrated that 34% of DEPs were related to targets of cognitive dysfunction in Model 1d after MI, 24% of DEPs were related to targets of adhesion spot in Model 3d after MI, and 29% of DEPs were related to targets of cognitive dysfunction in Model 7d after MI. The relationship between the DEPs and core targets of brain diseases was formed (Fig. 6B). These DEPs in the brain cortex established a relationship with the key proteins of neuroinflammation, such as Amyloid Beta precursor protein (APP), Apolipoprotein E (APOE), and Phosphatase and tensin homolog (PTEN). Neuroinflammation occures in the pathogenesis of TBI, AD, and PD. Amyloid beta (Aβ) peptides are the major component of amyloid plaques in AD, which are formed from the progressive cleavage of APP by beta- and gamma-secretase^36^. Studies have demonstrated that Aβ metabolism and aggregation are partly dependent on APOE, and the interaction between Aβ and APOE promotes Aβ deposition, which in turn contributes to memory deficit^37^.

The DEPs of the highest proportion were shown and classified by function and also by the biological process in which they were engaged in the LV and the brain cortex (Fig. 6C,D). The relationship of these DEPS is shown in the PPI plot (Fig. 6A, the right one). Including Complement and coagulation cascade proteins: C3-beta-c(C3), C4b-binding protein alpha chain(C4BPA), and Complement factor properdin (CFP) detected in LV. Mitochondrial translation-related protein: Mitochondrial ribosomal protein S14(MRPS14) was detected in LV and mitochondrial 39S ribosomal protein L22 (MRPL22) was detected in the brain cortex. Iectin protein Calreticulin (CALR). Cell adhesion-related proteins: Integrin beta (ITGB3) and Fibronectin (FN1) were detected in LV, Integrin alpha 3 variant A (ITGA3) was detected in the brain cortex, and Versican core protein (VCAN) was both detected in LV and brain cortex. TYROBP causal network-related protein Macrophage-capping protein (CAPG) was detected in LV and brain cortex. MAPK cascade-related protein Mitogen-activated protein kinase (MAPK11), myelin sheath-related protein Myelin basic protein (MBP), intracellular trafficking related protein Ras-related protein Rab-28(RAB28) and cell growth and survival protein Tyrosine-protein kinase receptor (IGF1R) detected in the brain cortex. Besides, ABCG4 is related to genes of cognitive dysfunction, adhesion spot, neuroinflammation, and energy metabolism in the brain cortex (Fig. 6D). More importantly, C-means cluster plot of DEPs in LV (Fig. S3A) and brain cortex (Fig. S3B) demonstrated that the expression level of DEPs is individual, and can be divided into several groups. DEPs in cluster 4, cluster 5, and cluster 6 have a similar expression mode in that more DEPs are activated and protein expression continues to rise over time in the LV. In the brain cortex, cluster 6 had a similar expression mode, indicating that the same amount of protein expression mode may reflect some common mechanisms shared in the heart and the brain. Among them, ELOVL5 was in cluster 5 in the LV and cluster 6 in the brain cortex. ABCG4 was in cluster 6 in the brain cortex. Therefore, ELOVL5 and ABCG4 were considered to perform the following study. According to the proteomics results, the protein expression of ELOVL5 and ABCG4 were gradually upregulated with time after MI (Fig. 6E,H). The WB results of ABCG4 and ELOVL5 in the brain cortex demonstrated that the expression of both ABCG4 and ELOVL5 was significantly increased when compared with the Sham group (Fig. 6F,G,I,J), and were consistent with the proteomic results.

## Discussion

The mortality due to complications of MI keeps increasing^1,38,39^. Among the unknown, complicated, and pathogenic factors, neuroinflammation is induced, such as microglia activation^26^, activation of the NF-κB pathway, and production of ROS in the brain^40^. However, the mechanism of neuroinflammation after MI is not clearly understood. Thus, in this study, a LAD ligation rat model was established, and markers of neuroinflammation were explored via IF to observe the neuroinflammation in the brain. Leukocyte infiltration occurred in the infarcted area of the LV as early as 1 day after MI. The collagen fibers colored blue occured and gradually increased with the prolongation of ischemia time in the heart. These results indicated that the morphology of the heart underwent a change due to ischemia and deteriorated as time progressed. This is consistent with the previously published results, which state that macrophages and neutrophils function as pro-inflammatory factors and repair by polarizing differentially from 1d after MI to 7d after MI^37,41^.

Neuroinflammation occurs in the brain induced by microglia according to studies^26^. However, there is no evidence available on when microglia are activated after MI. Indrajeetsinh Rana reported that activated microglia arise in the paraventricular nucleus (PVN) of the hypothalamus in the brain of rats at 2 weeks after MI, and Dworak observed that the activated microglia were located in the PVN 12 weeks after MI surgery in rats^42^. A study by Ferdinand Althammer demonstrated heart failure (HF)-induced microglial changes in the PVN 8 weeks post-MI surgery^43^. PVN is one of the neurosecretory nuclei. Yu Wang demonstrated that the macrophage‐inducible c‐type lectin receptor located in the PVN is a crucial target that affects sympathetic hyperactivity and mediates malignant ventricular arrhythmias post-MI^44^. James T. Thackeray reported that activation of microglia occurred as early as day 7 post-permanent ligation and microglia is activated again in the 8th-week post-MI in mice^26^. Our result demonstrated similar results. There was a significant increase in the quantity of microglia in the brain cortex compared with the Sham group as early as 1d post-MI in rats, and the number of IBA-1 positive cell kept an equivalent expression level at 3d post-MI and 7d post-MI (Fig. 2F). The phenomenon that the microglia in the brain cortex react immediately implies that MI does affect brain activity through a certain link. It has been shown that patients who have a history of MI have a higher risk of developing ischemic stroke and depression^1,2,45^, and people have impaired cognitive function in the early stages following HF^39^. Previous studies have demonstrated that neuroinflammation in the brain occurs followed by myocardial ischemia/reperfusion. This in turn leads to poor learning and memory via impairment of neurogenesis and long-term inflammation^46^.

To investigate the possible mechanism of neuroinflammation after MI, the expression of DEPs in the LV and brain cortex at 1d after MI, 3d after MI, and 7d after MI was analyzed based on DIA quantitative proteomics. Protein expression changed at different time points in the LV and brain cortex. The signature protein and biomarker, associated with MI, fibrosis, ROS generation, and inflammation were observed in the LV. NPPA belongs to the natriuretic peptide family^47,48^ and is used as cogent diagnostic indicators of MI and HF with other members such as BNP and NT-proBNP^49,50^. The initiation of NPPA helps to promote the establishment of pathological cardiac hypertrophy^51^. The expression of NPPA remains elevated at 1d post-MI, 3d after MI, and 7d after MI. This suggested that the myocardial injury worsens over time. The immune system is activated and monocytes play an important role in cardiac repair after MI, especially in the early stage. A correlation is seen between monocyte levels and the severity of cardiac dysfunction^52^. A quick accumulation of CD14 cells that originated from the spleen in the human myocardium mediates myocardial repair after MI^53,54^. Proteomics results from our study also demonstrated that CD14 is upregulated and continues to rise for a week following MI. HMGBs are released upon inflammation activation, subsequently stimulating immune responses as signaling molecules^55^. Elevated serum concentrations of HMGB2 exacerbate ischemic injury in MI by promoting the production of ROS, leading to cell apoptosis, inflammation, and reduced ejection fraction in MI patients^56^. NCAM1, also known as CD56, facilitates cellular adhesion in the injured heart, thereby contributing to TGFβ1- induced structure alterations and cardiac dysfunction^57^. The expression of EFHD2 is specifically increased in cardiomyocytes within the LV after 4 and 7 days post-MI during cardiac remodeling, repair, and spontaneous apoptosis through regulation of BCL2L1 abundance^58^, this upregulation was also observed at 3 days and 7 days post-MI according to our data. LOX significantly contributes to adverse effects on cardiac remodeling during post-infarction recovery strategies while promoting myocardial dysfunction^59^. ANXA1 exhibits pro-angiogenic properties by inducing macrophage polarization towards a pro-repair phenotype with high secretion levels of VEGF-A, thus facilitating neovascularization and cardiac repair processes^60^. Overexpression of TMEM214 promotes apoptosis by recruiting procaspase 4 to the endoplasmic reticulum^30^.

**Figure 7:**
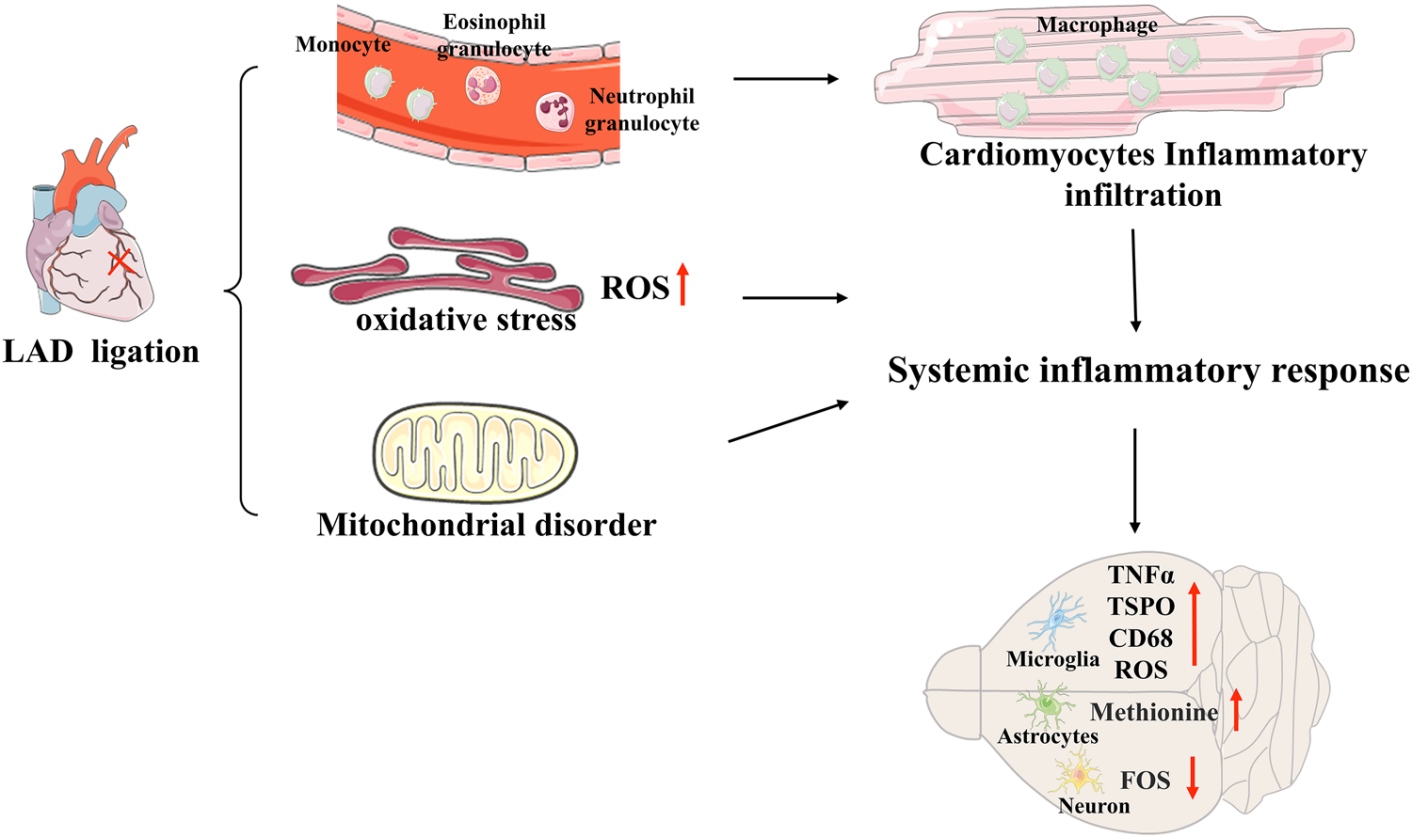
The possible mechanisms between the heart and brain after MI.

The development of MF occurs gradually with an increase in ischemia time. The extracellular matrix (ECM) primarily consists of fibrillar collagens, including COL5A1 and COL5A2. A proteomic analysis comparing scar tissue to normal heart tissue revealed robust expression of COL5A1 early in the scar formation during MI. Knocking out the COL5A1 gene resulted in an increased surface area and abnormal architecture of the scar, leading to a deterioration in heart function^61^. Repression of COL5A2 biosynthesis led to ECM reorganization, which plays a crucial role in vascular remodeling processes. The relationship between core targets of brain diseases and DEPs in the brain cortex was investigated to identify key proteins from the list of DEPs in the LV and the brain cortex. Through bioinformatics analysis, two highly relevant candidate proteins, ELOVL5 and ABCG4, were identified as functioning in the brain. ELOVL5, a member of the ELO family, is involved in the biosynthesis of long-chain polyunsaturated fatty acids, which are essential for maintaining proper myelin structure and the normal action potential conduction^62^. Studies have shown that decreased levels of C18 are associated with ELOVL5 in patients with complex MI^63^. Furthermore, researchers have identified the crucial role of ELOVL5 in olfactory sense and motor performance as well as its contribution to maintaining brain structure integrity^64^. In addition, ELOVL5 plays an indispensable role in supporting the normal structure of myelin for efficient electrical signal transmission along myelinated nerves within the peripheral nervous system^62^. ABCG4 belongs to the ABC transporter G-subfamily, which is essential in keeping lipid homeostasis in the cells and transporting drugs^65,66^. It was observed specifically in the brain and eye by Susan Oldfield^67^. Afterward, Masanori Tachikawa detected ABCG4 only in the grey matter of the mouse brain^68^. This finding aligns with the results of our study that ABCG4 was up-regulated in the brain cortex of rats after MI. In addition, ABCG4 also contributes significantly to neuro-systemic diseases. Aβ concentration and phosphorylated Tau theory represent two key mechanisms underlying AD pathogenesis ^69,70^. Yoshinari Uehara was the first to demonstrate a significant increase in ABCG4 expression in microglia within AD brain^71^. ABCG2 and ABCG4 mediate the efflux of amyloid-β peptide from the mouse brain across the blood–brain barrier^72^, thereby contributing partially to elevated cerebral Aβ concentrations. Various pathological processes contribute to neuroinflammation in a complex and multifactorial manner (Fig. 7). The systemic inflammatory response is one of the primary factors, involving inflammatory infiltration of macrophages primarily derived from monocytes in peripheral blood during acute stages^37^, endoplasmic reticulum OS induced by excessive ROS production^44^, and mitochondrial disfunction, all of which collectively induce neuroinflammation within the brain^7^. This is characterized by increased TSPOs within CNS^26^, upregulated expression of CD68 and TNFα (pro-inflammatory markers), overproduction of ROS localized in microglia, neuron deficits^40^, as well as elevated methionine levels within astrocytes^45^.

## Conclusions

The results of our study demonstrated that the number of microglia increased significantly in the brain cortex as early as 1d after MI and kept increasing within a week after MI, which may provide a reference for more appropriate intervention time after MI. To study the proteins after MI, differential proteome profiles of the LV and brain cortex were analyzed 1d after MI, 3d after MI, and 7d after MI based on DAI proteomics. Through bioinformatics analysis, we obtained a series of DEPs that may be related to the heart–brain interaction, including ELOVL5 and ABCG4. This article can provide a reference to someone who devoted themselves to studying the heart–brain interaction. However, more studies are needed to prove the relationship between the heart and brain.

## Materials and methods

### Rat surgery

A total of 83 Sprague Dawley male rats (280 − 320 g, 8 weeks old) were purchased from the SPF (Beijing) Biotechnology Co, Ltd. All the animals were housed under standard laboratory conditions (22°C, 40–50% humidity, 12/12 light/dark cycle) and had free access to food and water. The experiments were approved by relevant guidelines and regulations of the China Academy of Chinese Medical Sciences' Administrative Panel on Laboratory Animal Care and performed under the institutional guidelines and ethics of the committee as part of the China Academy of Chinese Medical Sciences (ERCCACMS21-2111-02). MI was accomplished by permanent ligation of the left anterior descending coronary artery (LAD) in this study. The rats were anesthetized with 1% pentobarbital sodium (Sigma, USA) via intraperitoneal injection. After performing an oral cavity endotracheal intubation, the heart was exposed and the LAD was permanently ligated using a 5–0 suture. The detailed operation was shown in the previous study^73^. Rats were kept warm until they woke up.LAD ligation rats were divided into four groups randomly, Model-1d group (n = 12), Model-3d group (n = 12), Model-5d group (n = 12), and MI-7d group (n = 12). Rats in the Sham group (n = 6) were subject to the same surgery except for the ligation. To ensure the ligation of LAD, electrocardiograph information was monitored and recorded during the surgery. After ligation for 1d, 3d, 5d, and 7d, the rats in the Model-1d group (n = 12), Model-3d group (n = 12), Model-5d group (n = 12), MI-7d group (n = 12) were sacrificed respectively in a different time, and the sham group was sacrificed after surgery of 7 days. The blood of the animals was collected from the abdominal aorta. The circulating blood was perfusion through the heart to remove circulating blood, and then the heart and the brain tissues were harvested for pathological staining or further analysis.

### Histopathological staining

Hematoxylin–eosin staining (H&E) staining can observe the damage of tissue slices, Masson attaining can detect the Cardiac fibrotic area, and for the Nissl bodies within neurons, Nissl staining is usually used. The heart and brain tissues were performed to HE staining. Firstly, sections were embedded in paraffin and cut into 4 μm-thick transverse sections. These sections were then processed further using the following steps: dewaxing to remove the paraffin and rehydration, hematoxylin dyeing solution, washing with running water, differentiated with differentiation liquid, washing with running water, using blue returning liquid to return blue, dehydration, and finally sealed the sections with neutral balsam. The images were observed using a microscope (Nikon Eclipse E100, Tokyo, Japan). The heart sections went through Masson staining according to the protocol (Servicebio, G1006, China). The heart sections were dewaxing as follows: Xylene I for 20 min; Xylene II for 20 min; 100% ethanol I for 5 min; 100% ethanol II for 5 min; 75% ethanol for 5 min; Rinsing with tap water. They were then soaked in Masson A to F solution as follows: Masson A overnight, and rinsed with tap water the other day; Masson solution composed of solution B and solution C in the ratio of 1:1 for 1 min, rinsed with tap water; immersed in 1% hydrochloric acid alcohol, rinsed with tap water; soaked in Masson D for 6 min, rinsed with tap water; Masson E for 1 min; Masson F for 2–30 s; rinsed with 1% glacial acetic acid and then dehydrated with two cups of anhydrous ethanol; 100% ethanol for 5 min; Xylene for 5 min; Finally sealed with neutral gum and observed using a microscope. For Nissl staining, the tissue slices were treated with Toluidine Blue for 5 min and rinsed with tap water after the brain sections were dewaxed. They were then treated with 0.1% Glacial acetic acid. Tap water was used to stop the reaction. After washing it with tap water, it was dried in the oven. The sections were immersed into xylene for 10 min, and sealed with neutral gum. Finally, the image was acquired with a microscope.

### Immunofluorescence (IF)

The expression level of IBA-1 was quantitatively measured by immunofluorescence to understand the neuroinflammation post-MI. After deparaffinization and rehydration, the brain slides were subject to heat-mediated antigen retrieval using EDTA antigen retrieval buffer (pH 9.0) for 15 min and 3% BSA to avoid non-specific binding. The brain sections were then incubated with rabbit anti-IBA-1 antibody (1:2000, ab178846, UK) in a wet box overnight at 4 °C. On the second day, the sections were incubated using AF594 (1:2000, ab150080, UK) for one hour at room temperature, the sections were then incubated with DAPI (1:1000, C0065, Solarbio, China) solution at room temperature for 10 min in darkness. Images were captured immediately by the Research Slide Scanner (VS120-S6- OLYMPUS, Japan). Five fields of each section in the cortex were randomly selected, and the part where IBA-1 overlaps with DAPI was considered to be microglia. The number of microglia was calculated to characterize the neuroinflammatory response.

### DIA proteomics

#### Total Protein Extraction

The heart and brain tissue were ground individually in liquid nitrogen and lysed with PASP lysis buffer (100 mM NH_4_HCO_3_, 8 M Urea, pH 8), followed by 5 min ultrasonication on ice. The lysate was centrifuged at 12,000 g for 15 min at 4 °C and the supernatant was reduced with 10 mM DTT for 1 h at 56 °C, and subsequently alkylated with sufficient iodoacetamide (IAM) for 1 h at room temperature in the dark. Then samples were completely mixed with 4 times the volume of precooled acetone by vortexing and incubated at – 20 °C for at least 2 h. Samples were centrifuged at 12,000×*g* for 15 min at 4 °C and the precipitation was collected. After washing with 1mL cold acetone, the pellet was dissolved by dissolution buffer (8 M Urea, 100 mM TEAB, pH 8.5). BSA standard protein solution was prepared according to the instructions of the Bradford protein quantitative kit. The standard curve was drawn with the absorbance of the standard protein solution and the protein concentration of the sample was calculated.

#### Trypsin treatment

Each protein sample was digested with trypsin at 37 °C for 4 h, and then trypsin and CaCl_2_ were added and digested overnight. Formic acid was mixed with digested sample to adjust pH under 3, and centrifuged at 12,000×*g* for 5 min at room temperature. The supernatant was slowly loaded to the C18 desalting column, washed with washing buffer (0.1% formic acid, 3% acetonitrile) 3 times, then added elution buffer (0.1% formic acid, 70% acetonitrile). The eluents of each sample were collected and lyophilized.

#### LC–MS/MS analysis

Evosep One UHPLC system (Evosep) coupled with an Orbitrap Q ExactiveTM HF-X mass spectrometer (Thermo Fisher) was applied in this study. A total of 4 μg fraction supernatant of each sample was separated via an analytical column (15 cm × 150 μm, 1.9 μm). Orbitrap Q ExactiveTM HF-X mass spectrometer performed with a spray voltage of 2.1 kV, Nanospray Flex™(ESI), and capillary temperature of 320 °C. Based on the DIA mode, the m/z range covered from 350 to 1500. MS1 resolution was set to 60,000 (at 200 m/z), the full scan AGC target value was 5 × 10^5^, and the maximum ion injection time was 20 ms. Peptides were fragmented by HCD in MS2, in which resolution was set to 30,000 (at 200 m/z), the AGC target value was 1 × 10^6^, and a normalized collision energy of 27%.

#### Protein identification and quantitation

The large class proteome database rattus_norvegicus_uniprot_2022_1_27.fasta.fasta(36,268 sequences) based on Uniport was used. The protein identification was performed using a Proteome Discoverer 2.2 (PD2.2, Thermo Fisher) with 10 ppm of mass tolerance. Oxidation of methionine (M) was specified as dynamic modification, and acetylation was specified as N-terminal modification in PD 2.2. A maximum of 2 missed cleavage sites were allowed. To obtain a reliable result, the identified protein contains at least 1 unique peptide. At least 2 peptide numbers for protein were considered a protein for quantitative analysis. The identified proteins were retained and performed with FDR < 1.0%. Spectronaut (version 14.0, Biognosys) software was used to obtain the quantitation result and the precursor ion Q-value cutoff was set to 0.01. Differentially expressed proteins (DEPs) were defined as the proteins whose quantitation was significantly different between the Model group and Sham group (*p* < 0.05 and FC≥ 1.5 or FC ≤ 0.5, [fold change, FC]). More details are shown in Table 1.

**Table 1:** Proteome discoverer analysis parameters.

Item	Value
Type of Quantification	Fragment Quantification
Enzyme	Trypsin
Max.Missed Cleavage Sites	2
Precursor Mass Tolerance	10 ppm
Fragment Mass Tolerance	0.02 Da
Dynamic Modification	Oxidation/ + 15.995 Da (M)
N-Terminal Modification	Acetyl/ + 42.011 Da (N-Terminal)
Static Modification	Carbamidomethyl/ + 57.021 Da (C)

#### GO and KEGG analyze

The DEPs in the LV and the brain cortex were subject to bioinformatics analysis. This included Gene Ontology (GO) and the Kyoto Encyclopedia of Genes and Genomes (KEGG). GO and KEGG were used to explore the function of DEPs in the LV and the brain cortex. The GO enrichment analysis included biological process (GO-BP), cellular component (GO-CC), and molecular function (GO-MF), all of which are based on the GO database (http://www.geneontology.org/). The pathway enrichment in the LV and the brain cortex was based on the KEGG database (https://www.kegg.jp/). Items with *p* < 0.05 are retained.

### Investigation of proteins that are related to brain diseases

All of the DEPs in the LV and the brain cortex were uploaded into the STRING online database (https://cn.string-db.org/). To investigate the relationship between DEPs in the LV and the brain cortex, a PPI of DEPs in the LV and the brain was formed. DEPs greater than the twofold degree median (twofold degree median = 22) were retained. For exploring the protein related to neuroinflammation, core genes of neuroinflammation, cognitive dysfunction, adhesion spot, and energy metabolism were obtained via the GeneCards database (https://www.genecards.org/) and only the first 25 genes were taken into consideration. The PPI network between the core gene of the disease and DEPs in the brain was analyzed.

### Western blotting

The LV tissue and brain cortex were thawed and the tissues were homogenized individually. The total protein was extracted using RIPA buffer and PMSF (Solarbio, R0010, China). The extracted total protein was centrifuged at 12,000×*g* rpm for 10 min at 4 ℃. The protein concentration of the supernatant was calculated by using the BCA Protein Assay Kit (Solarbio, PC0020, China). For Western blotting (WB), the protein concentration was normalized by using the SDS-PAGE loading buffer, 5 × (Gene-protein link, P06M18, China), and the RIPA buffer. The samples were loaded in the 10%SDS-PAGE Gels and proteins were then transferred from the gel to 0.45 μm polyvinylidene difluoride (PVDF) membranes (Thermo Fisher, US). The PVDF membrane was incubated on a shaker in 5% nonfat dry milk for 1 h at room temperature for blocking. It was then incubated overnight at 4 ℃ using the following primary antibodies (1:800, ABCG4, 14269-1-AP, US) (1:1000, ELOVL5, bs-7054R, China), and the membrane was then washed and incubated with the following secondary antibody (1:10,000, Goat Anti-Rabbit IgG H&L (HRP), ab6721, UK). The protein band was turned up by using ECL WB substrate, the results were measured using ImageJ (1.53K).

### Statistical analysis

Quantitative analysis of the images was done using GraphPad Prism (Version 9.0.0, GraphPad, USA). One-way analysis of variance (ANOVA) and multiple comparison testing was performed using the Bonferroni method to determine the statistical significance between the Sham and Model groups. *P* value < 0.05 was considered to be statistically significant.

### Ethical approval

All the animal experiments were performed by the ARRIVE guidelines, approved by the local animal care committee, and were under the ERCCACMS21-2111–02.

### Supplementary Information

Supplementary Information 1.

Supplementary Figure 1.

Supplementary Information 3.

Supplementary Information 4.

## Data Availability

The original contributions presented in the study are included in the article.

## Abbreviations

MI: Myocardial infarction
LV: Left ventricular
HF: Heart failure
MF: Myocardial fibrosis
AD: Alzheimer's disease
PD: Parkinson's disease
TSPO: Translocator protein
DIA: Data-independent acquisition
LAD: Left anterior descending
H&E: Hematoxylin-eosin
IF: Immunofluorescence
DEP: Differentially expressed protein
LC-MS/MS: Liquid chromatography-mass spectrometry/mass spectrometry
IAM: Iodoacetamide
GO: Gene ontology
KEGG: Kyoto encyclopedia of genes and genomes
BP: Biological process
CC: Cellular component
MF: Molecular function
WB: Western blotting
PVDF: Polyvinylidenedifluoride
ECG: Electrocardiogram
IBA-1: Ionized calcium-binding adaptor molecule-1
PCA: Principal component analysis
OS: Oxidative stress
ROS: Reactive oxygen species
NPPA: Atrial natriuretic peptide
CD14: Monocyte differentiation antigen
HMGB2: High mobility group protein B2
C8B: Complement component C8 beta chain
C4BPA: C4b-binding protein alpha chain
PTGIS: Prostacyclin synthase
NCAM1: Neural cell adhesion molecule 1
ORM1: Alpha-1-acid glycoprotein
CCDC22: Coiled-coil domain-containing protein 22
ANXA1: Annexin A1
CYBB: Cytochrome b-245 beta chain
MT2A: Metallothionein
TMEM214: Transmembrane protein 214
G6PDX: Glucose-6-phosphate 1-dehydrogenase
MGST1: Glutathione transferase
MICAL1: [F-actin]-monooxygenase MICAL1
SLC25A24: Solute Carrier Family 25 Member 24
COL5A1: Collagen alpha-1(V) chain
COL5A2: Collagen alpha-1(V) chain
COL18A1: Collagen type XVIII alpha 1 chain
COL8A1: Collagen type VIII alpha 1 chain
LOX: Protein-lysine 6-oxidase
CASP8: Caspase-8
EFHD2: EF-hand domain-containing protein D2
LCP1: Lymphocyte cytosolic protein 1
INPP5D: Phosphatidylinositol-3,4,5-trisphosphate 5-phosphatase
CAPG: Macrophage-capping protein
VCAN: Versican core protein
HP: Haptoglobin
DAB2: Disabled homolog 2
FTL1: Ferritin light chain 1
SNAP23: Synaptosomal-associated protein 23
PLCH1: Phosphoinositide phospholipase C
SPCS3: Signal peptidase complex subunit 3
RBP1: Retinol-binding protein 1
ALB: Albumin
ELOVL5: Elongation of very long chain fatty acids protein 5
APP: Amyloid Beta precursor protein
APOE: Apolipoprotein E
PTEN: Phosphatase and tensin homolog
Aβ: Amyloid beta
C3: C3-beta-c
C4BPA: C4b-binding protein alpha chain
CFP: Complement factor properdin
MRPS14: Mitochondrial ribosomal protein S14
MRPL22: Mitochondrial 39S ribosomal protein L22
CALR: Calreticulin
ITGB3: Integrin beta
FN1: Fibronectin
ITGA3: Integrin alpha 3 variant A
VCAN: Versican core protein
CAPG: Capping protein
MAPK11: Mitogen-activated protein kinase
MBP: Myelin sheath-related protein Myelin basic protein
RAB28: Ras-related protein Rab-28
IGF1R: Tyrosine-protein kinase receptor
PVN: Paraventricular nucleus
ECM: Extracellular matrix
