# Supplementary material for: Proteomic study of left ventricle and cortex in rats after myocardial infarction
Source: Sci Rep. 2024 Mar 22;14:6866. doi: 10.1038/s41598-024-56816-6 (PMC10958002; doi:10.1038/s41598-024-56816-6)

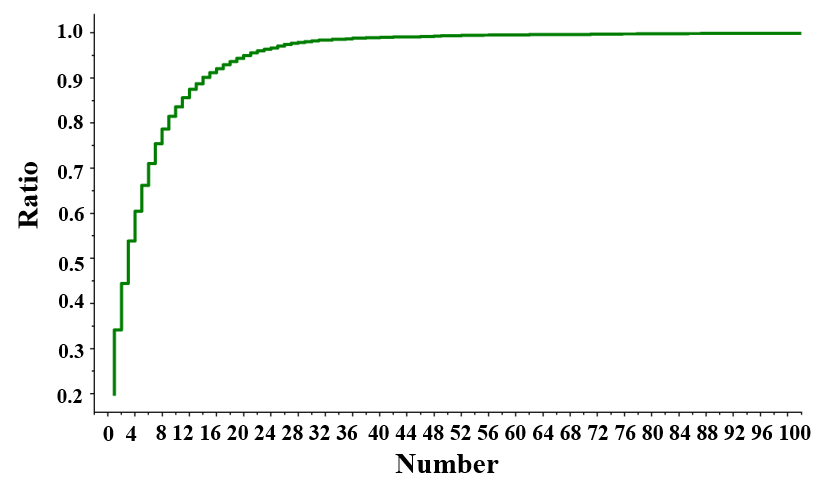


Fig 1. Distribution plot of unique peptide numbers in identified proteins: The abscissa represents the number of Unique peptides. The ordinate represents the cumulative proportion of the proteins containing the unique peptides to the total proteins as there is an increase in the number of Unique peptides. Unique peptides increase and more reliable proteins are identified when the curve increases more slowly.


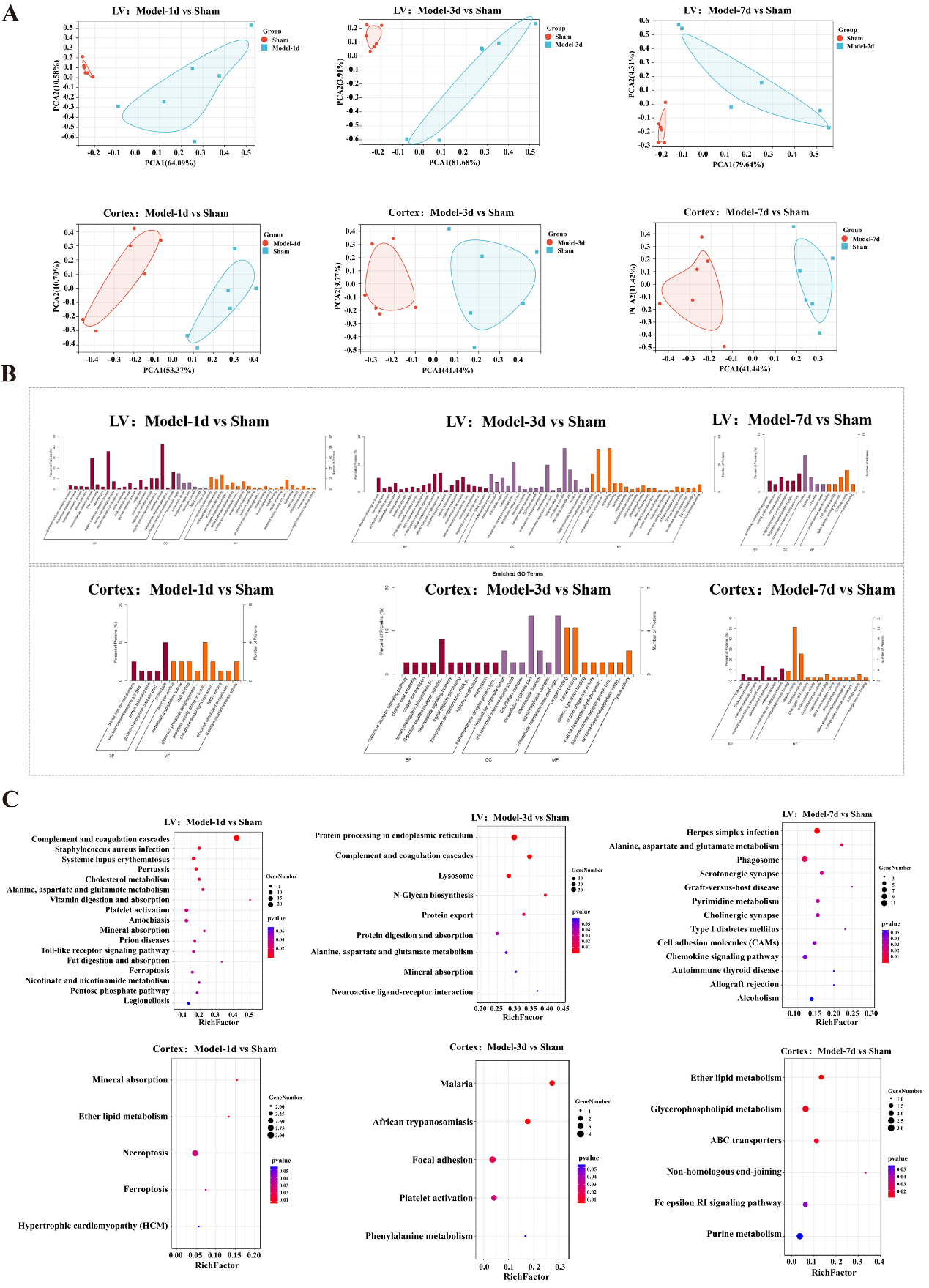


Figure 2. Differentially expressed protein in LV and brain cortex.

(A) Principal coordinates analysis (PCA) and consensus clustering in LV and brain cortex.

(B) Gene Ontology of differential proteins in LV and brain cortex at different time points after AMI. (C) KEGG pathways enriched by DEPs in LV and brain cortex (entries of p<0.05 were retained).


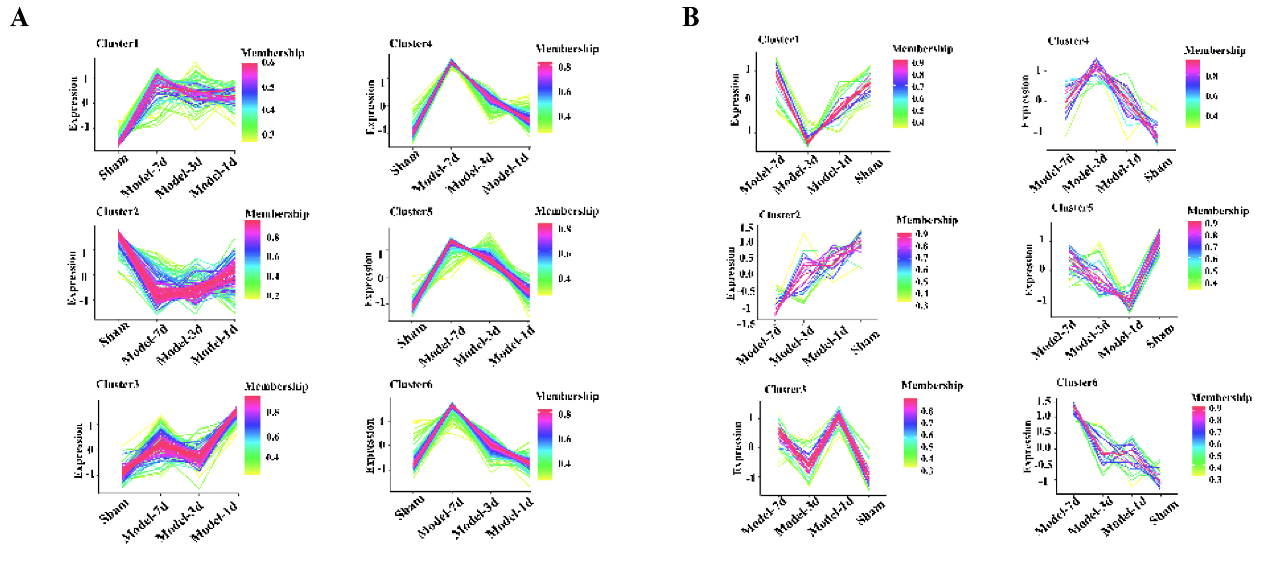


Fig.3 C-means Cluster Plots of DEPs in the LV and brain cortex.

(A) C-means Cluster Plot of DEPs in the LV. Based on their expression level, the differential proteins are separated into several classes. The abscissa represents the grouping, and the ordinate represents the corrected Z value of the expression level, the larger the ordinate value, the higher the expression level, and vice versa represents the lower expression level. Each broken line represents 1 protein, and the color represents the size of the value, with a larger value representing the closer the protein is to the average level in that classification. (B) C-means Cluster Plot of DEPs in the brain cortex.

Table 1. Cognitive impairment-related proteins (top 25 from Genecards).

| Number | Gene Symbol | Description | Relevance score |
| --- | --- | --- | --- |
| 1 | APOE | Apolipoprotein E | 53.74 |
| 2 | SCN8A | Sodium Voltage-Gated Channel Alpha Subunit 8 | 44.29 |
| 3 | MAPT | Microtubule Associated Protein Tau | 43.41 |
| 4 | APP | Amyloid Beta Precursor Protein | 36.03 |
| 5 | BDNF | Brain Derived Neurotrophic Factor | 32.91 |
| 6 | COMT | Catechol-O-Methyltransferase | 32.19 |
| 7 | PSEN1 | Presenilin 1 | 29.98 |
| 8 | SNCA | Synuclein Alpha | 28.41 |
| 9 | GJB2 | Gap Junction Protein Beta 2 | 28.04 |
| 10 | FOXP1 | Forkhead Box P1 | 28.02 |
| 11 | FMR1 | FMRP Translational Regulator 1 | 26.26 |
| 12 | GNB5 | G Protein Subunit Beta 5 | 26.26 |
| 13 | PTEN | Phosphatase and Tensin Homolog | 26.12 |
| 14 | SCN1A | Sodium Voltage-Gated Channel Alpha Subunit 1 | 26.10 |
| 15 | MTHFR | Methylenetetrahydrofolate Reductase | 25.37 |
| 16 | PRNP | Prion Protein | 24.21 |
| 17 | MECP2 | Methyl-CpG Binding Protein 2 | 23.37 |
| 18 | NALCN | Sodium Leak Channel, Non-Selective | 23.17 |
| 19 | TNF | Tumor Necrosis Factor | 22.96 |
| 20 | SPG11 | SPG11 Vesicle Trafficking Associated, Spatacsin | 22.74 |
| 21 | NEFL | Neurofilament Light Chain | 22.43 |
| 22 | USH2A | Usherin | 22.32 |
| 23 | NOTCH3 | Notch Receptor 3 | 22.23 |
| 24 | TP53 | Tumor Protein P53 | 22.15 |
| 25 | GRIN2A | Glutamate Ionotropic Receptor NMDA Type Subunit 2A | 21.95 |

Table 2. Adhesion plaque-related proteins (top 25 from Genecards).

| Number | Gene Symbol | Description | Relevance score |
| --- | --- | --- | --- |
| 1 | ITGB2 | Integrin Subunit Beta 2 | 98.47 |
| 2 | FERMT3 | FERM Domain Containing Kindlin 3 | 63.45 |
| 3 | ICAM1 | Intercellular Adhesion Molecule 1 | 61.68 |
| 4 | VCAM1 | Vascular Cell Adhesion Molecule 1 | 49.04 |
| 5 | SELE | Selectin E | 39.77 |
| 6 | PTK2 | Protein Tyrosine Kinase 2 | 37.36 |
| 7 | PSEN1 | Presenilin 1 | 35.08 |
| 8 | APP | Amyloid Beta Precursor Protein | 34.23 |
| 9 | APOE | Apolipoprotein E | 33.64 |
| 10 | SELP | Selectin P | 33.10 |
| 11 | PECAM1 | Platelet And Endothelial Cell Adhesion Molecule 1 | 32.75 |
| 12 | ICAM2 | Intercellular Adhesion Molecule 2 | 31.74 |
| 13 | ITGAL | Integrin Subunit Alpha L | 31.61 |
| 14 | ITGA4 | Integrin Subunit Alpha 4 | 30.90 |
| 15 | NCAM1 | Neural Cell Adhesion Molecule 1 | 30.88 |
| 16 | ITGB1 | Integrin Subunit Beta 1 | 28.64 |
| 17 | SELL | Selectin L | 28.61 |
| 18 | CEACAM1 | CEA Cell Adhesion Molecule 1 | 27.05 |
| 19 | TNF | Tumor Necrosis Factor | 26.65 |
| 20 | CTNNB1 | Catenin Beta 1 | 26.32 |
| 21 | DSP | Desmoplakin | 26.02 |
| 22 | JUP | Junction Plakoglobin | 25.83 |
| 23 | ITGAM | Integrin Subunit Alpha M | 24.00 |
| 24 | PXN | Paxillin | 23.95 |
| 25 | EPCAM | Epithelial Cell Adhesion Molecule | 23.83 |

Table 3. Energy metabolism-related proteins (top 25 from Genecards).

| Number | Gene Symbol | Description | Relevance score |
| --- | --- | --- | --- |
| 1 | MTR | 5-Methyltetrahydrofolate-Homocysteine Methyltransferase | 47.23 |
| 2 | MMACHC | Metabolism of Cobalamin Associated C | 47.12 |
| 3 | MTRR | 5-Methyltetrahydrofolate-Homocysteine Methyltransferase Reductase | 46.87 |
| 4 | INS | Insulin | 45.81 |
| 5 | LEP | Leptin | 44.63 |
| 6 | CYP2D6 | Cytochrome P450 Family 2 Subfamily D Member 6 | 39.65 |
| 7 | PPARG | Peroxisome Proliferator Activated Receptor Gamma | 38.86 |
| 8 | ABCA3 | ATP Binding Cassette Subfamily A Member 3 | 36.43 |
| 9 | CYP2C19 | Cytochrome P450 Family 2 Subfamily C Member 19 | 34.87 |
| 10 | GHRL | Ghrelin and Obestatin Prepropeptide | 34.43 |
| 11 | IL6 | Interleukin 6 | 33.75 |
| 12 | ADIPOQ | Adiponectin, C1Q and Collagen Domain Containing | 33.02 |
| 13 | TPK1 | Thiamin Pyrophosphokinase 1 | 32.01 |
| 14 | CYP3A4 | Cytochrome P450 Family 3 Subfamily A Member 4 | 31.02 |
| 15 | MMADHC | Metabolism Of Cobalamin Associated D | 30.60 |
| 16 | UCP3 | Uncoupling Protein 3 | 28.38 |
| 17 | PPARGC1A | PPARG Coactivator 1 Alpha | 28.35 |
| 18 | SFTPC | Surfactant Protein C | 28.15 |
| 19 | ALB | Albumin | 27.94 |
| 20 | APOE | Apolipoprotein E | 27.91 |
| 21 | LEPR | Leptin Receptor | 27.38 |
| 22 | MTHFR | Methylenetetrahydrofolate Reductase | 27.31 |
| 23 | GCK | Glucokinase | 26.88 |
| 24 | MMAA | Metabolism Of Cobalamin Associated A | 26.74 |
| 25 | INSR | Insulin Receptor | 26.56 |

Table 4. Neuroinflammation related proteins (top 25 from Genecards).

| Number | Gene Symbol | Description | Relevance score |
| --- | --- | --- | --- |
| 1 | STAT2 | Signal Transducer and Activator of Transcription 2 | 7.13 |
| 2 | PTGS2 | Prostaglandin-Endoperoxide Synthase 2 | 4.37 |
| 3 | NRROS | Negative Regulator of Reactive Oxygen Species | 3.49 |
| 4 | APP | Amyloid Beta Precursor Protein | 3.07 |
| 5 | TSPO | Translocator Protein | 2.83 |
| 6 | SPHK1 | Sphingosine Kinase 1 | 2.66 |
| 7 | TNF | Tumor Necrosis Factor | 2.54 |
| 8 | IL1B | Interleukin 1 Beta | 2.48 |
| 9 | IL10 | Interleukin 10 | 2.48 |
| 10 | SNCA | Synuclein Alpha | 2.24 |
| 11 | NLRP3 | NLR Family Pyrin Domain Containing 3 | 2.02 |
| 12 | NR1D1 | Nuclear Receptor Subfamily 1 Group D Member 1 | 1.88 |
| 13 | APOE | Apolipoprotein E | 1.85 |
| 14 | CCL2 | C-C Motif Chemokine Ligand 2 | 1.84 |
| 15 | TLR4 | Toll Like Receptor 4 | 1.82 |
| 16 | AGER | Advanced Glycosylation End-Product Specific Receptor | 1.74 |
| 17 | IL6 | Interleukin 6 | 1.71 |
| 18 | NOS2 | Nitric Oxide Synthase 2 | 1.70 |
| 19 | MAPT | Microtubule Associated Protein Tau | 1.61 |
| 20 | CHI3L1 | Chitinase 3 Like 1 | 1.51 |
| 21 | TREM2 | Triggering Receptor Expressed on Myeloid Cells 2 | 1.50 |
| 22 | MMP3 | Matrix Metallopeptidase 3 | 1.50 |
| 23 | CX3CL1 | C-X3-C Motif Chemokine Ligand 1 | 1.47 |
| 24 | PECAM1 | Platelet And Endothelial Cell Adhesion Molecule 1 | 1.47 |
| 25 | MMP9 | Matrix Metallopeptidase 9 | 1.47 |

MI-1d


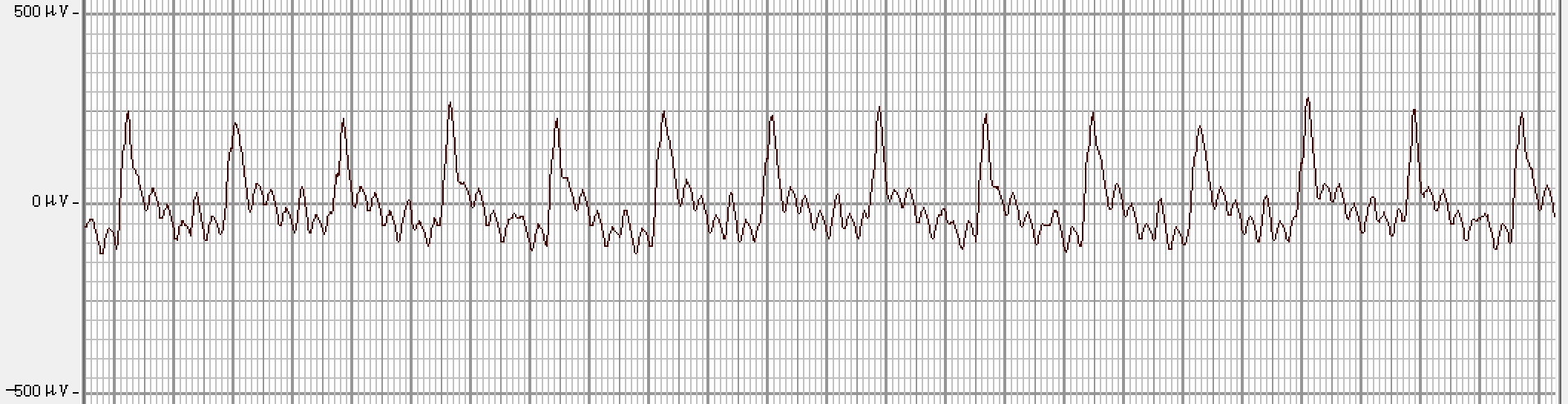

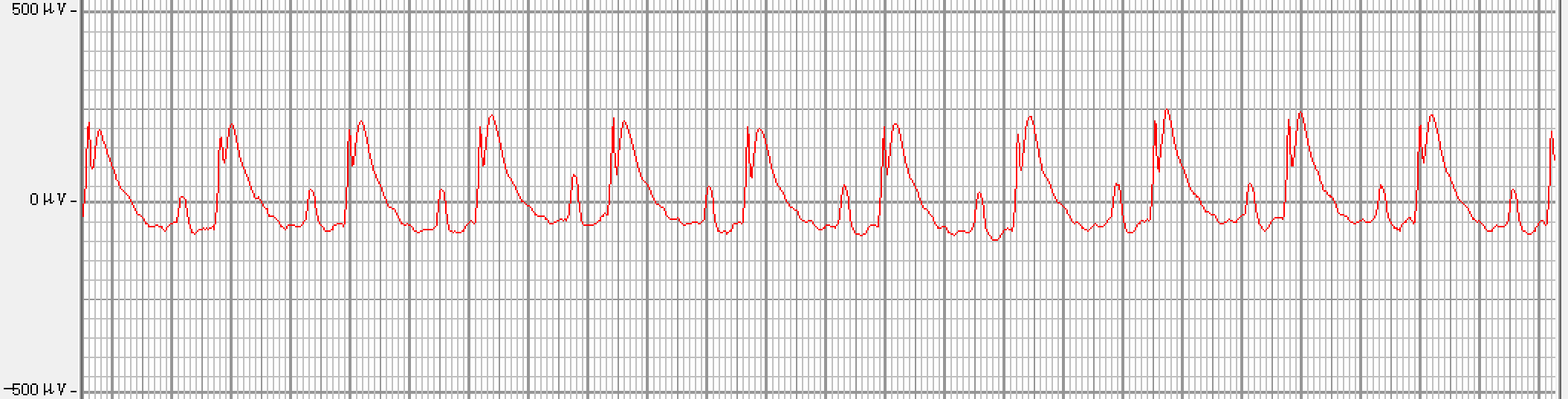

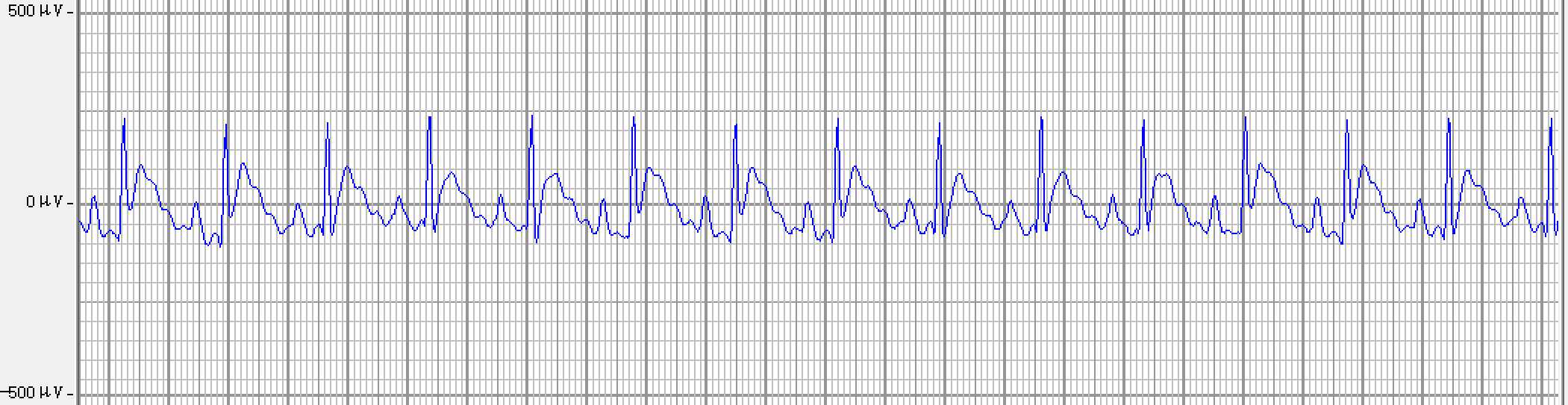

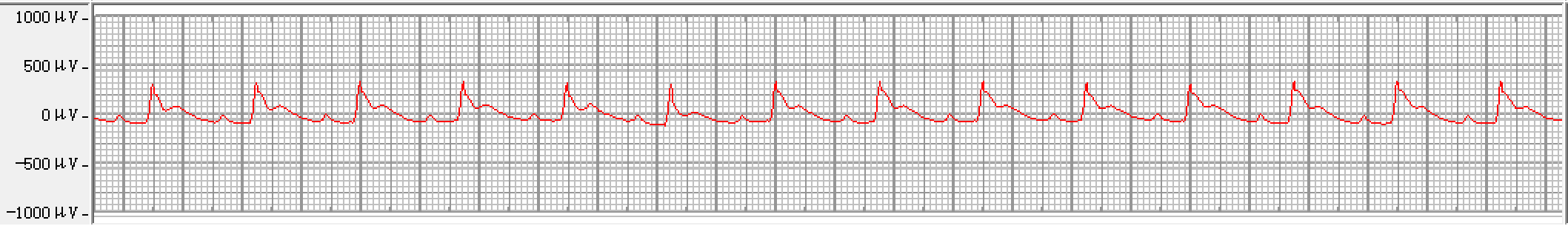

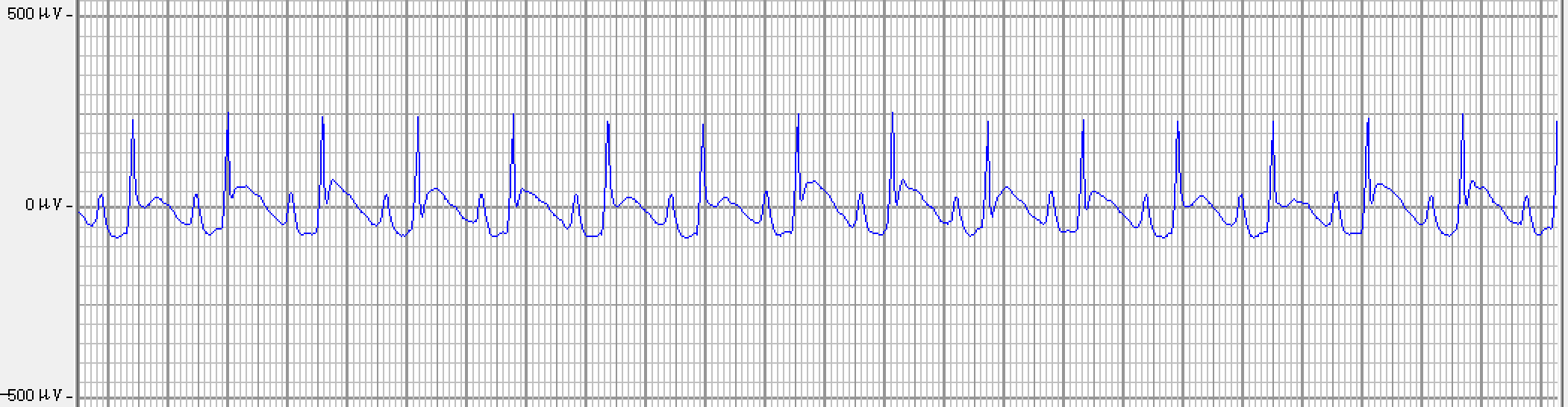

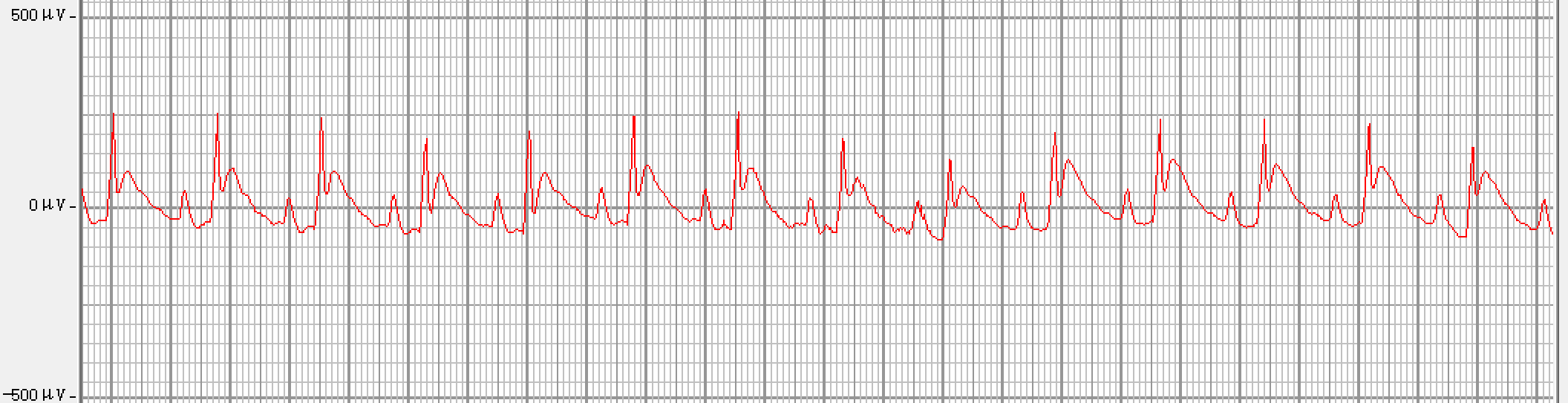


MI-3d


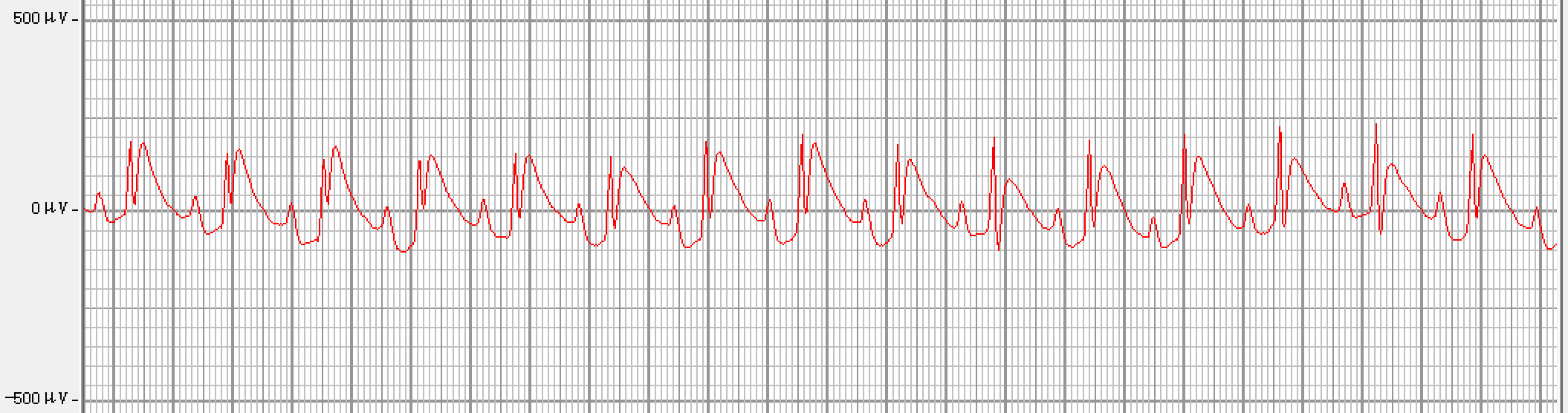

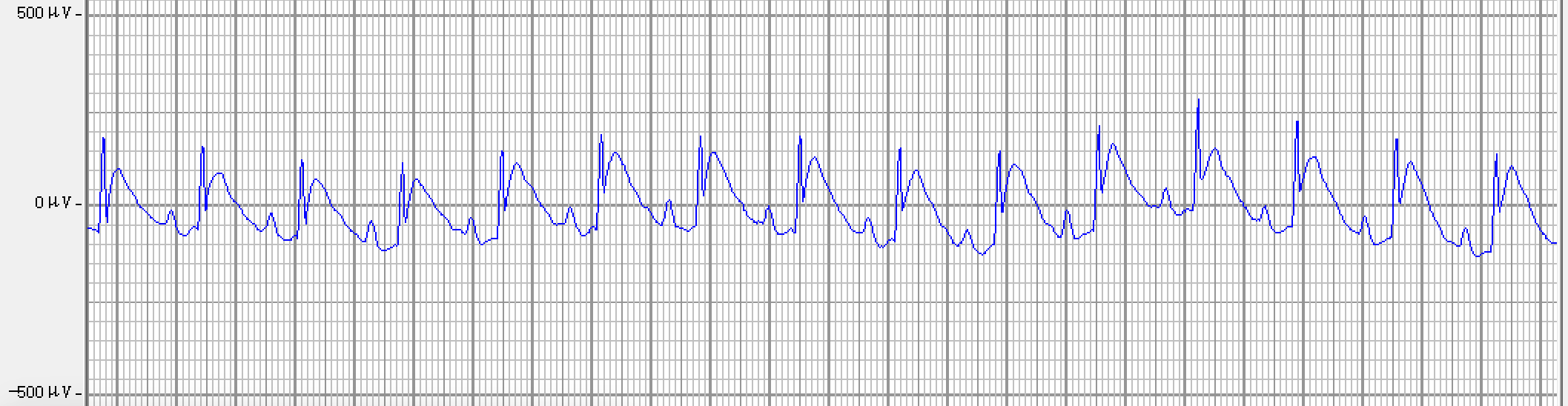

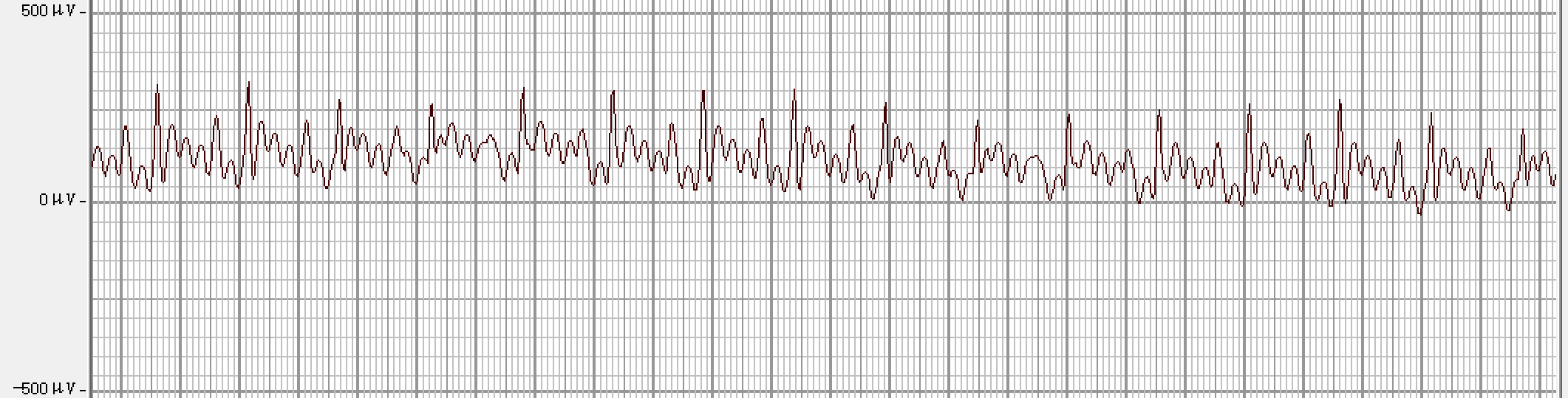

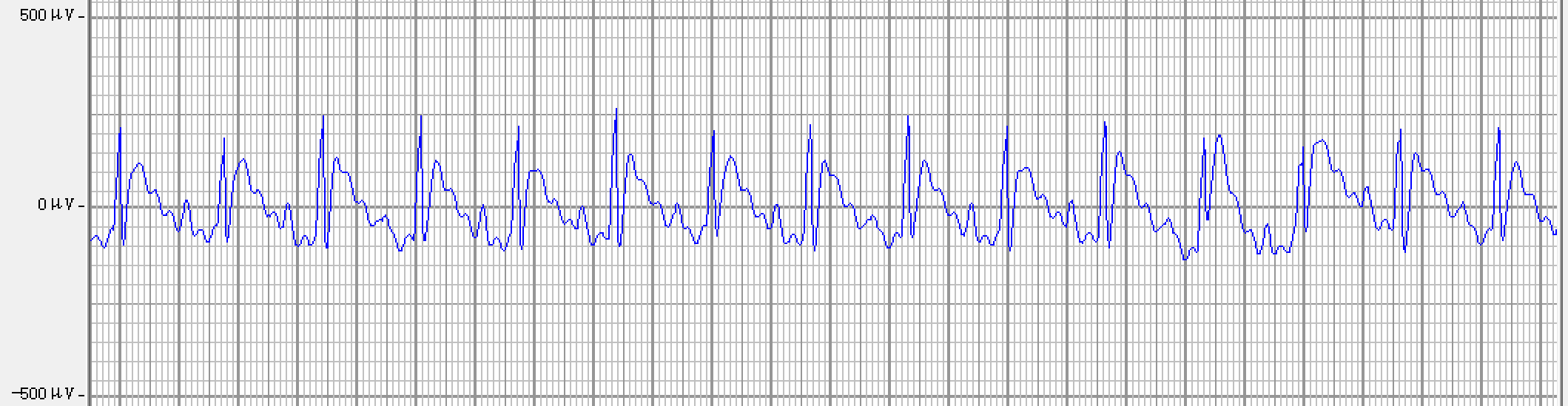

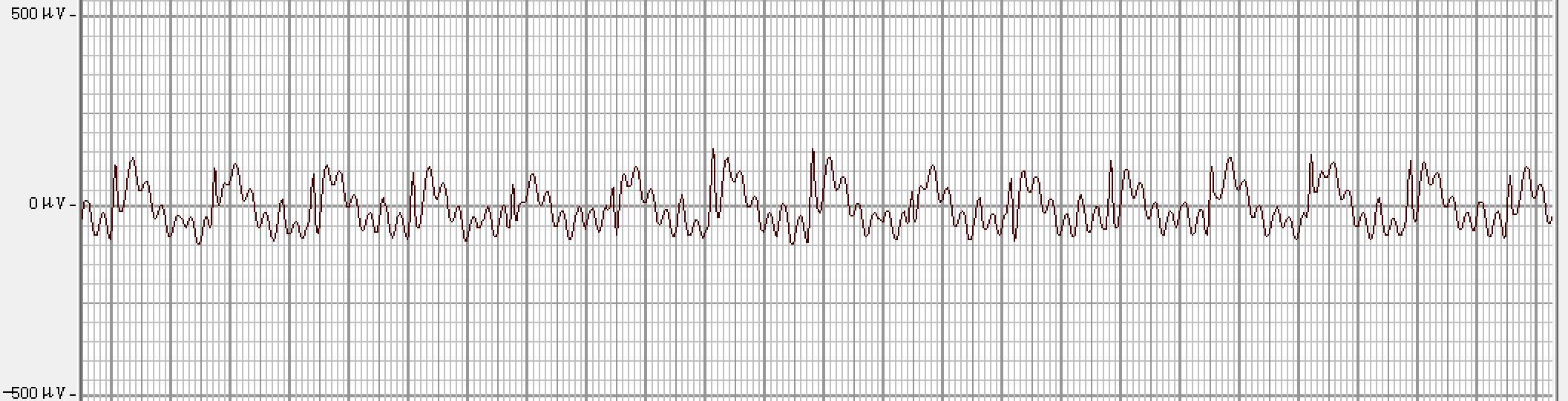

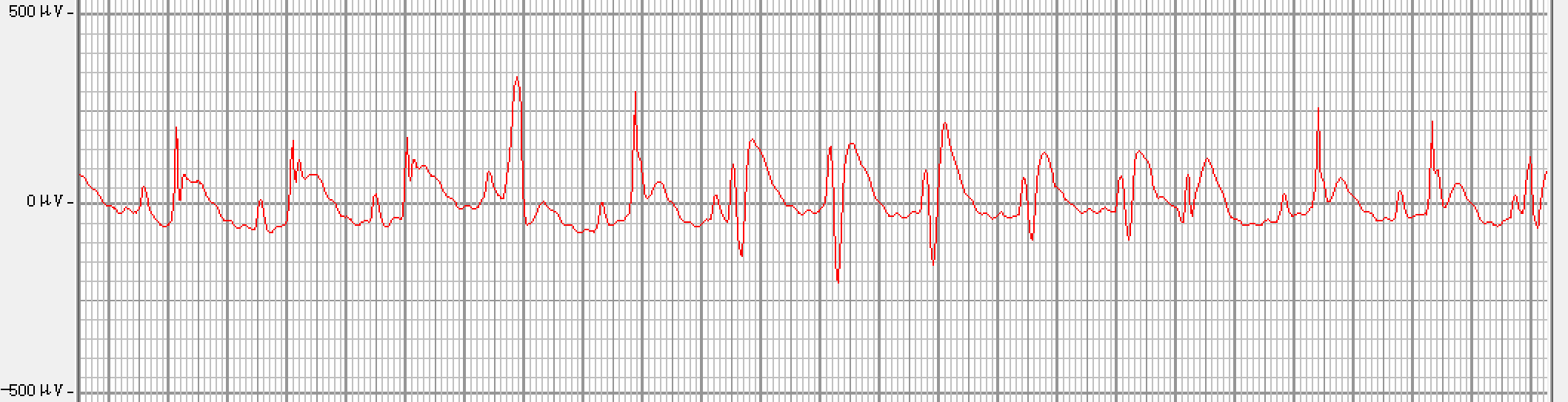


MI-7d


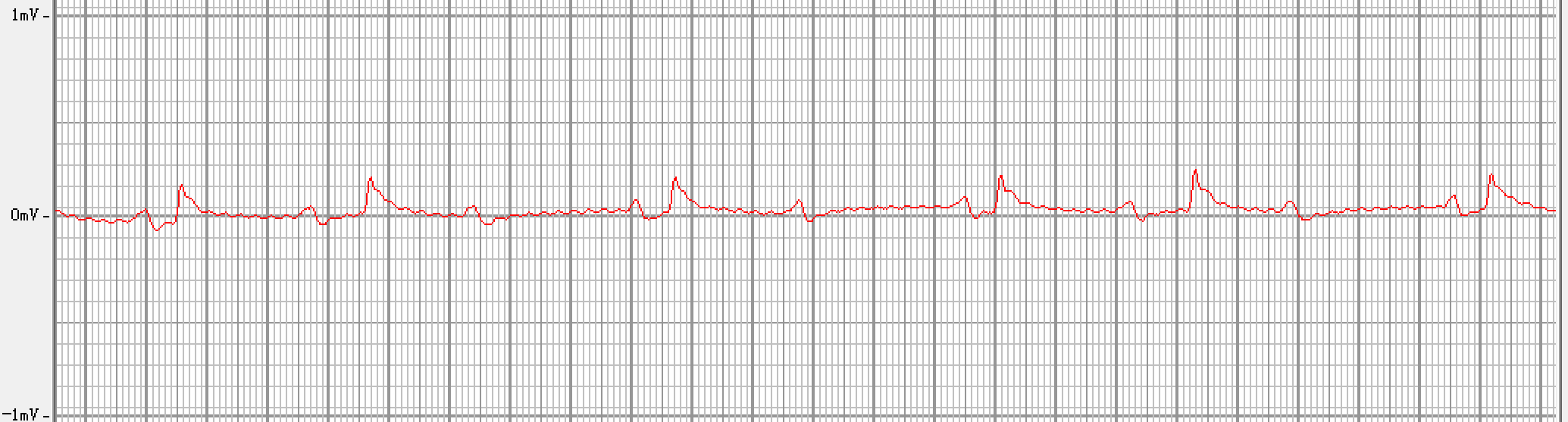

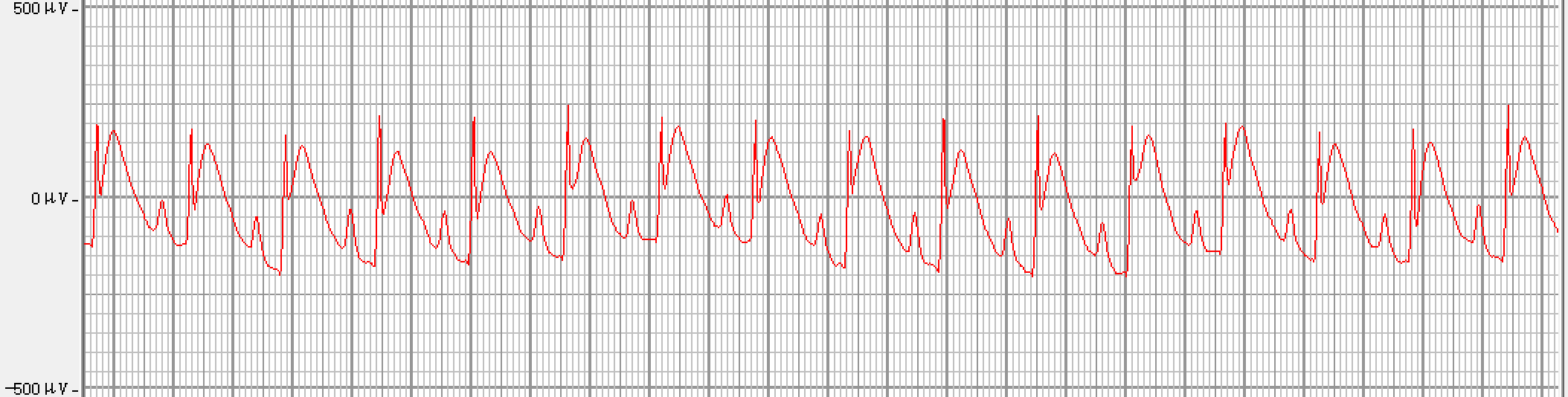

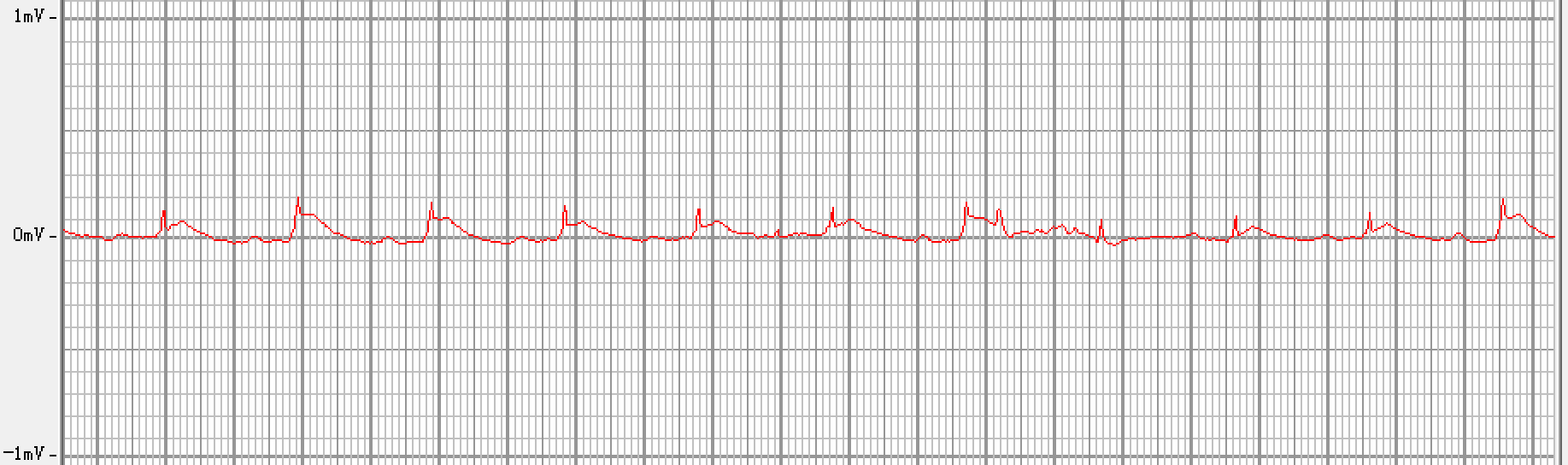

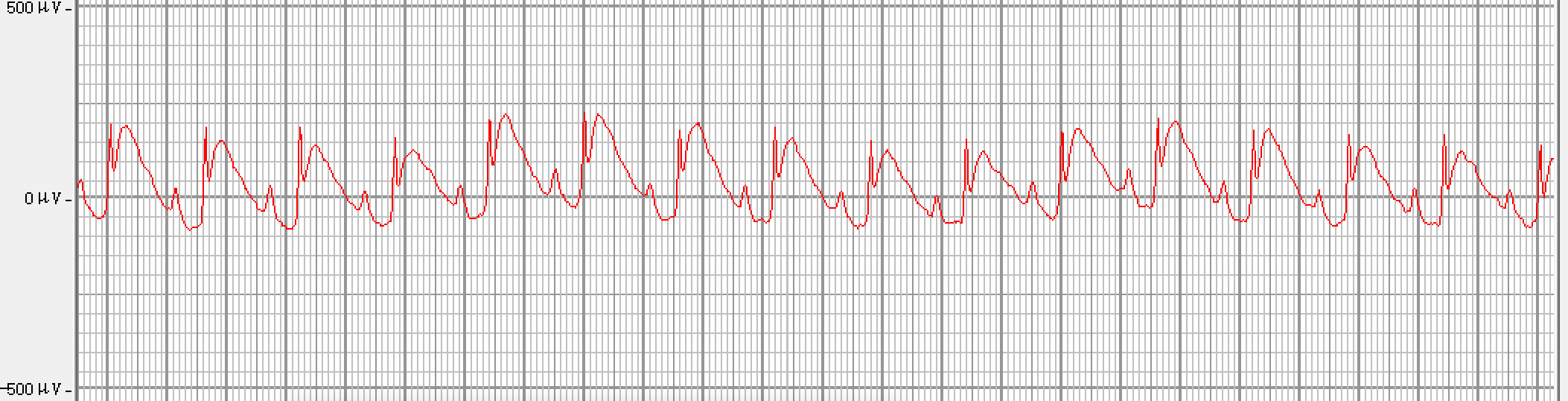

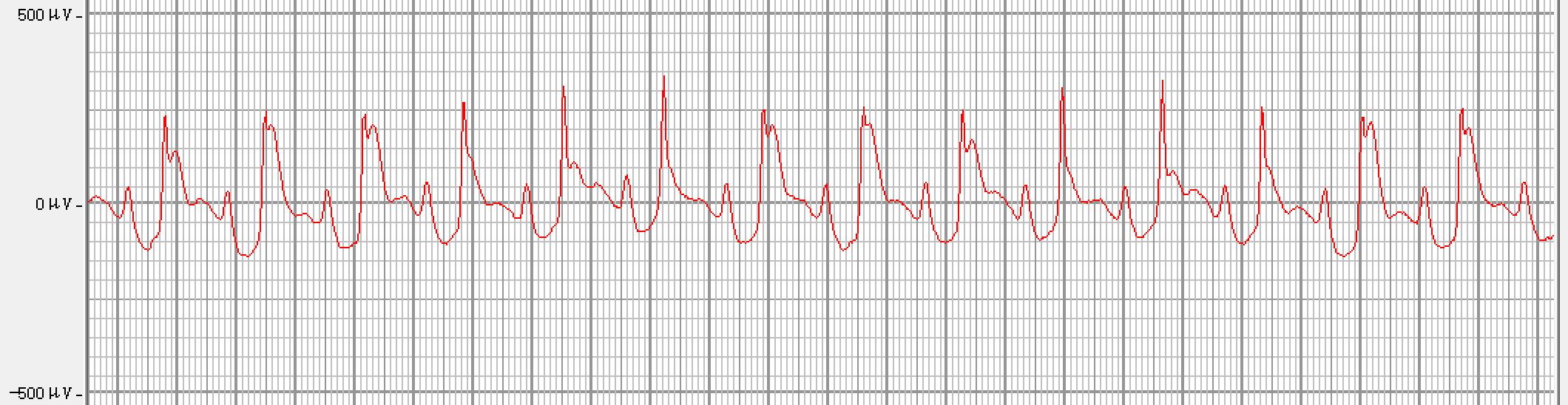

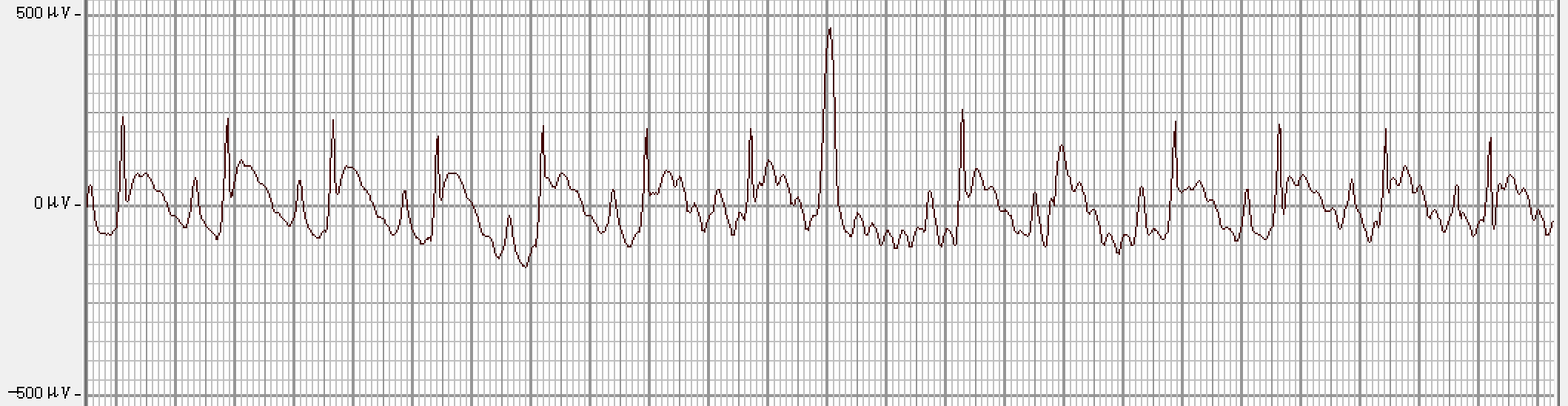

Supplement: Supplementary file 1 — Supplementary Information 1. [file 41598_2024_56816_MOESM1_ESM.docx]
