# Supplementary material for: Proteomic study of left ventricle and cortex in rats after myocardial infarction
Source: Sci Rep. 2024 Mar 22;14:6866. doi: 10.1038/s41598-024-56816-6 (PMC10958002; doi:10.1038/s41598-024-56816-6)
Supplement: Supplementary file 2 — Supplementary Figure 1. [file 41598_2024_56816_MOESM2_ESM.docx]

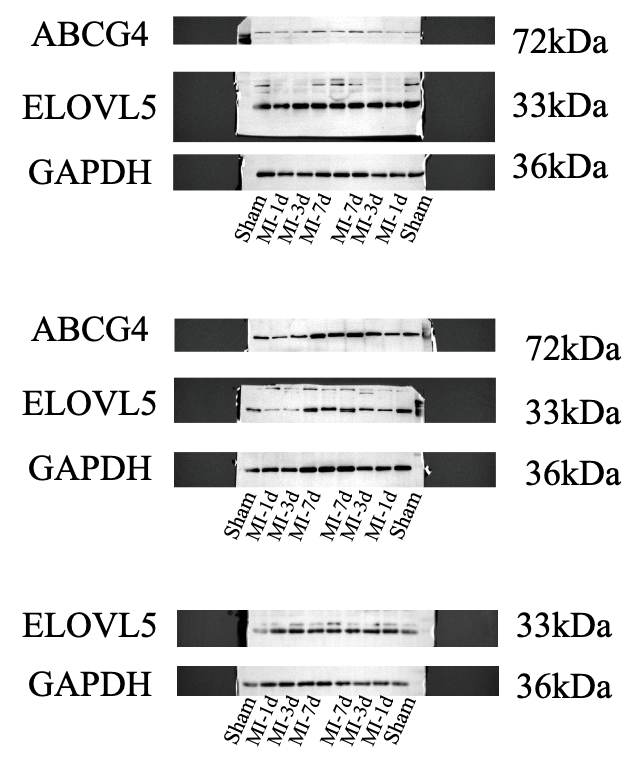


Fig.1 All WB results.

Note: The middle channel is for a sample of another group, which is not covered by this article, so it is not included in the statistics.
